# GAHIB: graph attention VAE with a hyperbolic information bottleneck for biologically structured single-cell representations

**DOI:** 10.3389/fgene.2026.1863100

**Published:** 2026-06-26

**Authors:** Zeyu Fu, Jiawei Fu, Xiaoxia Wang, Yiyao Liu, Tianfei Ran

**Affiliations:** 1 State Key Laboratory of Trauma and Chemical Poisoning, Institute of Combined Injury, Chongqing Engineering Research Center for Nanomedicine, College of Preventive Medicine, Army Medical University, Chongqing, China; 2 Department of Orthopedics, Xinqiao Hospital, Army Medical University, Chongqing, China; 3 Department of Hepatobiliary Surgery, The Second Affiliated Hospital of Chongqing Medical University, Chongqing, China; 4 State Key Laboratory of Trauma and Chemical Poisoning, Key Laboratory of Medical Protection for Electromagnetic Radiation, Ministry of Education of China, Institute of Toxicology, College of Preventive Medicine, Army Medical University, Chongqing, China

**Keywords:** graph attention network, hyperbolic geometry, information bottleneck, representation learning, single-cell RNA-seq, variational autoencoder

## Abstract

Current single-cell RNA-sequencing (scRNA-seq) variational autoencoders (VAEs) usually emphasise local cell graph structure, hyperbolic latent geometry, or bottleneck compression separately, yet these biases are rarely evaluated together in one evidence-gated representation model. We present GAHIB (graph attention VAE with a hyperbolic information bottleneck), which combines a graph-attention encoder, a 2D information bottleneck, and a Lorentz-hyperbolic geometry loss. Because the main clustering benchmark uses Leiden-derived proxy labels rather than definitive biological ground truth, we evaluate the model in two tiers: a broad 53-dataset proxy-label benchmark for method characterisation, and curated-label and marker analyses on annotated systems for biological interpretation. Across the proxy benchmark, GAHIB shows a balanced aggregate profile across clustering, projection-quality, and latent-structure metrics, while important comparisons remain mixed: scVI is statistically close to NMI/ARI, and scDHMap remains competitive on DRE-UMAP. On curated-label systems, the biological signal remains context-dependent; muscle atlas analyses support lineage-aligned structure with marker enrichment, whereas the fine T-cell immune-subtype task favors scVI. Sensitivity, seed-stability, a bounded count-dropout pilot, and cost analyses indicate practical runtime under the tested settings; however, the dropout evidence is limited to named pilot systems, and complete manually curated provenance remains an explicit limitation. Together, the results position GAHIB as a complementary, geometrically aware, single-cell representation rather than a drop-in clustering replacement.

## 1 Introduction

Single-cell RNA-sequencing (scRNA-seq) now routinely resolves transcriptional state at individual-cell resolution, yielding high-dimensional count matrices with thousands of genes per cell (Zheng et al., 2017). Many downstream analyses of these data—clustering into cell types, inferring trajectories, detecting differential expression, and annotating states—depend on the *latent representation* used to summarise each cell. Widely adopted pipelines such as Scanpy (Wolf et al., 2018) and Leiden clustering (Traag et al., 2019) inherit the strengths and weaknesses of whichever embedding is fed into them, so the choice of latent space is has consequence far beyond the embedding step itself (Luecken and Theis, 2019; Lähnemann et al., 2020).

Unlike bulk expression measurements, scRNA-seq matrices are sparse, over-dispersed, and well approximated by negative-binomial count models (Hafemeister and Satija, 2019; Svensson, 2020). They are also structurally heterogeneous: discrete cell identities coexist with continuous transitional states and branching developmental programmes that standard clustering alone cannot resolve (Trapnell et al., 2014; Haghverdi et al., 2016). An embedding useful in practice must, therefore, satisfy several requirements simultaneously: it must denoise counts, preserve local neighbour relations for clustering and graph construction, and retain enough global organisation for reliable trajectory inference (Saelens et al., 2019) and biological interpretation. Methods that optimise only one of these goals can produce either clean clusters with poor manifold structure or smooth manifolds with weak cell-state separation.

Variational autoencoders (VAEs) have emerged as a widely used framework for single-cell representation learning (Lopez et al., 2018; Grønbech et al., 2020). scVI (Lopez et al., 2018) and its semi-supervised extension scANVI (Xu et al., 2021) exemplify this paradigm, fitting a negative-binomial likelihood with a multi-layer perceptron (MLP) encoder to yield Euclidean embeddings suitable for downstream clustering. These models are effective denoisers, but their default Euclidean latent spaces still treat neighbourhood continuity and lineage branching as secondary effects rather than explicit structural constraints. Despite widespread adoption, standard VAEs may leave two structural priors under-specified. First, cell types can form *differentiation hierarchies* (stem → progenitor → differentiated), a branching topology that can be difficult to represent in Euclidean geometry without distortion—hyperbolic space embeds tree-like structures with exponentially lower distortion (Nickel and Kiela, 2017; Ganea et al., 2018). Second, cells form *local neighbourhoods* in gene-expression space, yet many VAE encoders treat each cell independently and do not directly use the cell graph topology.

**FIGURE 1:**
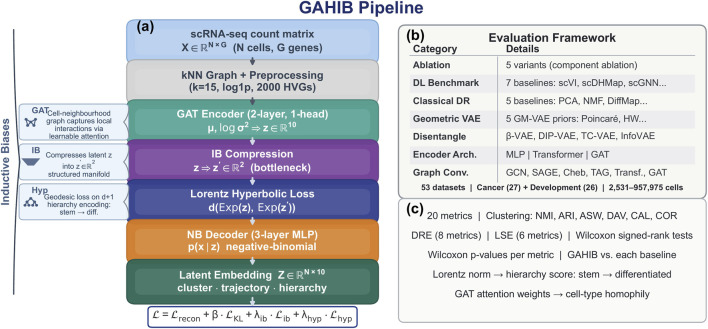
Overview of GAHIB. **(a)** GAHIB combines graph attention, an information bottleneck (IB), and Lorentz-hyperbolic regularisation to learn biologically structured embeddings from single-cell RNA sequencing (scRNA-seq) data. **(b)** Raw counts are converted into a k-nearest-neighbour (kNN) cell graph after highly variable gene (HVG) selection, encoded by a graph attention network (GAT) into a 10-dimensional latent space, and decoded with a negative-binomial (NB) head. **(c)** Evaluation spans 53 scRNA-seq datasets, 11 study tracks, and 20 metrics, including dimensionality-reduction evaluation (DRE), latent-space evaluation (LSE), UMAP-based projection quality, and Wilcoxon signed-rank tests.

Recent methods have begun to address these limitations, although only individually. Hyperbolic VAEs and related geometric latent-variable models (Mathieu et al., 2019; Nagano et al., 2019) explore non-Euclidean priors; scGNN (Wang et al., 2021) and scGCC (Tian S.-W. et al., 2023) build graph-aware encoders; scDHMap (Tian T. et al., 2023) maps latent codes onto the Poincaré disk; and siVAE (Choi et al., 2023) extracts gene-level interpretability via decoder Jacobians. The deep variational information bottleneck (Alemi et al., 2017) provides a variational framework for structured compression yet has rarely been coupled to hyperbolic geometry in single-cell settings. Thus, these ingredients are still usually addressed separately. GAHIB brings graph-structured encoding, information-bottleneck compression, and hyperbolic geometry together within one generative framework (Figure 1).

In this article, we propose **GAHIB**, which integrates three structural components into a single VAE.

1. **Graph attention network (GAT) encoder.** A two-layer GAT operates on a k -nearest-neighbour cell graph—a learning attention-weighted neighbourhood aggregation that captures cell-type-aware interactions.
2. **Information bottleneck (IB) layer.** A linear compression maps the d -dimensional latent code z to a 2D manifold coordinate $z^{\prime }$ , creating a structured low-dimensional representation amenable to geometric losses.
3. **Lorentz hyperbolic geometry loss.** Geodesic distance on the Lorentz hyperboloid between z and $z^{\prime }$ encourages the latent space to develop hierarchical organisation, with progenitor-like states expected to occupy lower-norm positions and differentiated states higher-norm positions.

A key architectural feature is that the IB layer supplies the compressed manifold coordinate $z^{\prime }$ required to define a stable target for the hyperbolic loss, while the GAT encoder strengthens both geometric terms through neighbourhood-aware representations.

We organize the analysis around an evidence hierarchy rather than a single leaderboard. One tier, curated/source-label and marker analyses, asks where the learned geometry aligns with non-Leiden biological labels or marker signatures. A second tier, the broad 53-dataset benchmark, characterises behaviour under a consistent Leiden proxy reference, which is useful for comparability but is not treated as biological ground truth. A third tier, robustness, sensitivity, and cost studies, states where the current evidence is complete and where it remains bounded by pilot settings or incomplete metadata.

We evaluate GAHIB through 11 experimental studies—7 comparative benchmarks and 4 robustness-and-efficiency studies—complemented by a biological-interpretation analysis. The complete original figure set is retained in the manuscript for traceability, while the text below consolidates the high-level biological and statistical claims. The seven *comparative benchmarks* quantify GAHIB’s performance relative to prior art.

- Component ablation (five variants, Section 5.2).
- Deep learning benchmark (eight methods, Section 5.3).
- Classical DR benchmark (six methods, Section 5.4).
- Geometric VAE benchmark (six methods, Section 5.5).
- Disentanglement comparison (six methods, Section 5.6).
- Encoder architecture comparison (three types, Section 5.7).
- Graph convolution sweep (six operators, Section 5.8).

A separate *biological-interpretation* analysis (Section 6) asks whether the learned latent space captures developmental hierarchy, gene programs, cell-type homophily, and tissue-specific Gene Ontology biological process enrichment (Section 6.6) on a representative subset of datasets for which cell-type or lineage annotations are available. Four *robustness and efficiency* studies (Section 7) further quantify latent-dimension stability (Section 7.1), multi-seed reproducibility (Section 7.1), computational cost and scaling (Section 7.2), and hyperparameter sensitivity (Section 7.4). Each experiment is paired with mean ± std performance estimates and Wilcoxon signed-rank tests against GAHIB, thus enabling consistent statistical comparison across study types.

## 2 Related research

### 2.1 Deep generative models for scRNA-seq

scVI (Lopez et al., 2018) introduced the VAE framework to single-cell analysis with a negative-binomial decoder. Subsequent methods extended this with contrastive learning (CLEAR—Han et al., 2022), deep adaptive clustering (scDAC—An et al., 2024), batch integration (SCALEX—Xiong et al., 2022), and graph-based encoders (scGNN—Wang et al., 2021; scGCC—Tian S.-W. et al., 2023). scDHMap (Tian T. et al., 2023) uses a hyperbolic decoder to preserve hierarchical distances but employs an MLP encoder and does not incorporate an information bottleneck.

### 2.2 Hyperbolic representations

Hyperbolic spaces embed tree-like hierarchies with exponentially lower distortion than Euclidean space (Nickel and Kiela, 2017). Ganea et al. (2018) extended this to hyperbolic neural networks operating on the Lorentz hyperboloid via the exponential map—the geometric foundation we adopt in GAHIB; hyperbolic representations have since proven effective in language settings (Poincaré GloVe—Tifrea et al., 2019) as well as in single-cell biology (Poincaré maps—Klimovskaia et al., 2020). Hyperbolic VAEs established Poincaré and wrapped-normal latent distributions (Mathieu et al., 2019; Nagano et al., 2019), while Poincaré maps and scDHMap adapted hyperbolic geometry to single-cell structure (Klimovskaia et al., 2020; Tian T. et al., 2023). These lines of research motivate the geometric-prior controls evaluated below, but they do not jointly optimise graph attention, a bottleneck coordinate, and Lorentz geometry in one single-cell variational model. GAHIB advances this by coupling a GAT encoder with an explicit IB → hyperbolic geometry pipeline, thereby enabling each component to reinforce the others.

### 2.3 Graph neural networks for single-cell data

Graph attention networks (Veličković et al., 2018) learn attention-weighted message passing, and several single-cell methods have used this idea to stabilise clustering on noisy cell graphs. scGAC introduced a graph-attentional clustering architecture for scRNA-seq cell embeddings (Cheng and Ma, 2022). SCEA combines an MLP encoder with a graph-attention autoencoder to jointly learn cell and gene embeddings for cell-type classification (Akbari Rokn Abadi et al., 2023). More recently, scVAG integrated a variational autoencoder with a graph-attention autoencoder for unified single-cell clustering (Laghaee et al., 2024). CellVGAE also uses graph attention networks for an unsupervised scRNA-seq VAE analysis framework (Buterez et al., 2022). GAHIB is positioned within this lineage as an integration of graph attention with an explicit information bottleneck and Lorentz-hyperbolic geometry loss; therefore, we avoid claiming superiority over graph-attention methods that were not included in the present benchmark.

### 2.4 Information bottleneck and disentanglement

The information bottleneck principle (Tishby et al., 2000) compresses representations to retain task-relevant information while discarding noise. Alemi et al. (2017) casted this as a variational objective (VIB), enabling end-to-end learning with reparameterised stochastic encoders. Disentanglement methods such as β -VAE (Higgins et al., 2017), DIP-VAE (Kumar et al., 2018), β -TC-VAE (Chen et al., 2018), FactorVAE (Kim and Mnih, 2018), and InfoVAE (Zhao et al., 2019) apply posterior regularisation to encourage statistically independent latent dimensions. Our empirical results indicate that these regularisers *decrease* clustering quality on scRNA-seq data, likely because they sacrifice reconstruction fidelity for disentanglement. GAHIB instead uses the IB layer as a *geometric* bottleneck—a structured anchor for hyperbolic distance losses—rather than a disentanglement objective.

## 3 Methods

### 3.1 Problem setting

Given a scRNA-seq count matrix $X\in R^{N\times G}$ with N cells and G genes, we aim to learn a latent representation $Z\in R^{N\times d}$ that preserves biological structure (cell types, trajectories, and hierarchies) while discarding technical noise.

### 3.2 Architecture overview

**FIGURE 2:**
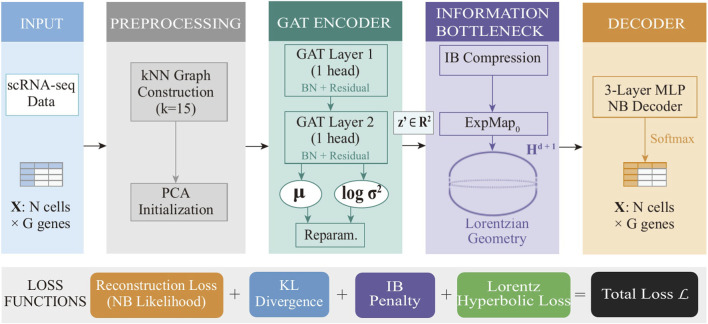
GAHIB architecture block diagram. Preprocessed counts are converted to a k -NN graph, encoded by a two-layer GAT, and sampled into a 10-dimensional latent code. The latent representation is then constrained by an information bottleneck, regularised in Lorentz-hyperbolic space, and decoded with a negative-binomial head. The objective combines reconstruction, KL, bottleneck, and hyperbolic losses.

GAHIB is a variational autoencoder comprising three integrated modules (Figure 2).

#### 3.2.1 GAT encoder

We construct a k -nearest-neighbour graph (k=15) over cells using Scanpy’s neighbourhood graph on a 50-D PCA embedding with Euclidean distance and UMAP-style connectivity weights. A two-layer GAT encoder (hidden dimension 128, single attention head, dropout 0.05) with BatchNorm, ReLU, and a residual link maps each cell $x_{i}$ to variational parameters $\left(\mu_{i},\log\sigma_{i}^{2}\right)$ via attention-weighted neighbourhood aggregation, as defined in Equation 1:

$$\mu_{i},\log\sigma_{i}^{2}=\text{GAT}\left(x_{i},{\left\{x_{j}\right\}}_{j\in N\left(i\right)}\right).$$ (1)

The latent code is sampled via the reparameterisation trick: $z_{i}=\mu_{i}+\sigma_{i}⊙\epsilon$ , ϵ∼N(0,I) .

#### 3.2.2 Information bottleneck

A linear encoder $W_{e}$ compresses $z\in R^{d}$ to a 2D manifold coordinate $z^{\prime }=W_{e}z\in R^{2}$ , and a linear decoder $W_{d}$ maps $z^{\prime }$ back to $R^{d}$ . The compressed coordinate $z^{\prime }$ is fed through the same negative-binomial decoder used for z , yielding an auxiliary reconstruction loss $L_{\text{ib}}=-\log p_{\text{NB}}\left(x|z^{\prime }\right)$ weighted by $\lambda_{\text{ib}}=0.5$ . Training $z^{\prime }$ to retain reconstruction-relevant information makes it a meaningful manifold coordinate; without this term, the bottleneck is untrained and the Lorentz distance below becomes degenerate.

#### 3.2.3 Lorentz hyperbolic geometry

Both z and $z^{\prime }$ are mapped to the Lorentz hyperboloid $L^{d}=\left\{x\in R^{d+1}:{\left\langle x,x\right\rangle }_{L}=-1,x_{0}>0\right\}$ via the exponential map at the origin. The hyperbolic geometry loss (Equation 2),

$$L_{\text{hyp}}=d_{L}\;\left(\text{ExpMap}\left(z\right),\;\text{ExpMap}\left(z^{\prime }\right)\right),$$ (2)

encourages hierarchical organisation by penalising geodesic distance on the hyperboloid between the full and compressed representations. The Lorentz norm $\|\text{ExpMap}\left(z_{i}\right){\|}_{L}$ provides a natural *hierarchy score*: cells with lower norms occupy positions closer to the hyperboloid origin (stem/progenitor), while those with higher norms are pushed towards the boundary (differentiated).

#### 3.2.4 NB decoder

An MLP decoder parameterises a negative-binomial likelihood $p\left(x_{i}|z_{i}\right)$ , capturing the overdispersion and library-size variation of count data.

### 3.3 Training objective

The total loss (Equation 3) combines four terms:

$$L=\underset{\text{NB likelihood}}{\underbrace{L_{\text{recon}}}}+\beta \cdot \underset{\text{regularisation}}{\underbrace{\text{KL}\left(q\left(z|x\right)\|p\left(z\right)\right)}}+\lambda_{\text{ib}}\cdot \underset{\text{bottleneck}}{\underbrace{L_{\text{ib}}}}+\lambda_{\text{hyp}}\cdot \underset{\text{geometry}}{\underbrace{L_{\text{hyp}}}},$$ (3)

with default weights β=0.1 , $\lambda_{\text{ib}}=0.5$ , and $\lambda_{\text{hyp}}=5.0$ . The low β permits flexible posterior approximation, while the strong $\lambda_{\text{hyp}}$ anchors hierarchical structure.

### 3.4 Subgraph sampling

Training the GAT encoder on the full cell graph is memory-intensive. We employ *subgraph sampling*: each epoch draws 10 subgraphs of 512 nodes from the full graph, providing sufficient gradient signal to match the number of mini-batch updates used by MLP encoders. Without this step, the graph encoder would receive only one gradient update per epoch versus ∼16 for the MLP, which would systematically undertrain the GAT and confound any fair comparison.

### 3.5 *Post hoc* interpretation analyses

We define four *post hoc* analyses that extract biological insight from a trained GAHIB model without retraining.

#### 3.5.1 Poincaré projection and Lorentz norm

Each latent code $z_{i}$ is mapped to the Lorentz hyperboloid via the exponential map, yielding $h_{i}={\text{ExpMap}}_{0}\left(z_{i}\right)\in L^{d+1}$ . The *Lorentz norm* $r_{i}=\cosh^{-1}\left(h_{i,0}\right)$ measures geodesic distance from the hyperboloid origin. We project to the Poincaré disk via the diffeomorphism $p_{i}=h_{i,1:d+1}/\left(1+h_{i,0}\right)$ , providing a 2D visualisation where the radial coordinate encodes hierarchical depth.

#### 3.5.2 Stemness score

We define a per-cell stemness score as the normalised inverse Lorentz norm: $s_{i}=1-\left(r_{i}-r_{\text{min}}\right)/\left(r_{\text{max}}-r_{\text{min}}\right)$ . The Spearman correlation $\rho_{s}$ between $s_{i}$ and external developmental annotations (when available) or Leiden cluster ordering quantifies the agreement between hyperbolic geometry and biological hierarchy.

#### 3.5.3 Decoder Jacobian gene attribution

For each cell i we compute the decoder Jacobian $J_{i}=\partial g\left(z_{i}\right)/\partial z_{i}\in R^{G\times d}$ , where g is the NB decoder mean function. Aggregating $\left|J_{i}\right|$ across a random subsample of 200 cells yields a gene × dimension importance matrix. Genes with high column norms in this matrix are the top attributed genes per latent dimension, representing learned gene programs. Marker overlap (MO) is computed as the fraction of differentially expressed marker genes (Wilcoxon rank-sum, Bonferroni-corrected p<0.05 ) that appear among the top-100 Jacobian-attributed genes.

#### 3.5.4 Lorentz-norm pseudotime

We use the Lorentz norm itself as an unsupervised pseudotime: $r_{i}=\cosh^{-1}\left(h_{i,0}\right)$ where $h_{i}={\text{ExpMap}}_{0}\left(z_{i}\right)$ . The cell with the smallest $r_{i}$ is taken as the developmental root. We validate $r_{i}$ against Scanpy diffusion pseudotime (DPT, computed from PCA →k NN → diffusion map with k=15 ) via Spearman and Kendall rank correlations. Two Euclidean baselines are included for comparison: Euclidean distance in 30-D PCA space and 2-D UMAP space, both from the same root cell.

#### 3.5.5 GO biological process enrichment

For each latent dimension $L_{k}$ , we compute the Pearson correlation between $L_{k}$ and every gene across all cells, rank genes by |ρ| , and take the top 100. We query these gene lists against a curated built-in panel of hallmark-style gene sets derived from MSigDB Biological Process categories (Ashburner et al., 2000) via a hypergeometric test in the spirit of gene-set enrichment analysis (Subramanian et al., 2005), applying Benjamini–Hochberg correction at adjusted p<0.05 . For the cross-dataset summary, we take the minimum adjusted p -value per term across datasets and report the top-three most significant terms per latent dimension.

#### 3.5.6 GAT attention homophily

We extract the learned attention coefficients $\alpha_{ij}$ from the final GAT layer for all edges (i,j) in the cell graph. Attention homophily is defined as the attention-weighted fraction of same-type neighbours: $H=\sum_{\left(i,j\right)}\alpha_{ij}\cdot 1\left[y_{i}=y_{j}\right]\;/\;\sum_{\left(i,j\right)}\alpha_{ij}$ , where $y_{i}$ is the Leiden cluster label of cell i . Values near 1 indicate that the GAT preferentially attends to biologically similar cells.

## 4 Experimental setup

### 4.1 Datasets

**FIGURE 3:**
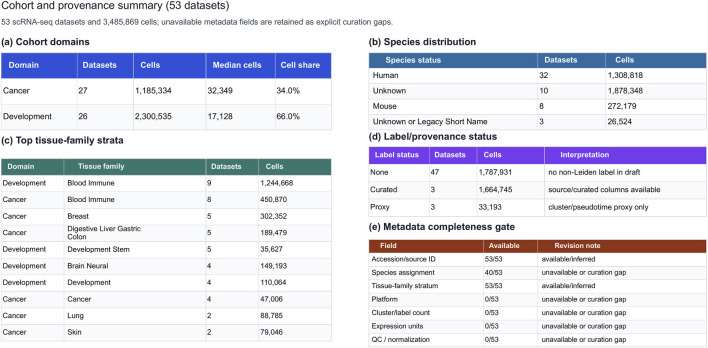
Structured cohort and provenance summary for the 53 scRNA-seq evaluation datasets. **(a)** Cohort domains report dataset counts, cell totals, median cell counts, and cell-share fractions. **(b)** Species assignment summarises the available or inferred species strata. **(c)** Leading tissue-family strata are shown by domain. **(d)** Label/provenance status distinguishes curated/source labels, proxy labels, and datasets without non-Leiden labels in the available metadata. **(e)** Metadata-completeness gate records provenance, indicating which fields are available and which remain explicit curation gaps.

We evaluated 53 scRNA-seq datasets spanning two biological domains (Figure 3).

- **Cancer** (27): skin (basal and squamous cell carcinoma), breast (six datasets, including epithelial, stromal, and metastatic), liver (hepatocellular, hepatoblastoma, and metastasis), blood/leukaemia (AML, ALL, multiple myeloma, and lymphoma), GI tract (gastric and colorectal), lung adenocarcinoma (two), brain metastasis (two), Merkel cell carcinoma (two), and T-cell cancers.
- **Development** (26): haematopoiesis (eight datasets from CD34^+^ progenitors to aged HSCs), neural development (dentate gyrus, spinal cord, retina, and astrocytes), embryonic/stem cell systems (hESC time series and endoderm), organ development (lung, pituitary, and teeth), disease models (Alzheimer’s and LPS response), and atlas-scale references (PanSci muscle and T-cell).

The datasets span both humans and mice, range from 2,531 to 957,975 cells pre-subsampling, and include both primary tumours and developmental trajectories, thus providing a comprehensive test of GAHIB’s generalisability across biological contexts. We also compiled a 53-row metadata inventory from the available manifests, recording accession or source identifier, cell and gene counts, broad domain, inferred species and tissue-family strata, and annotation availability. Fields that were not recoverable from the existing manifests—platform, original measurement units, source cluster counts, and detailed QC/normalisation provenance—are marked as unavailable rather than inferred.

All datasets are preprocessed identically: library-size normalisation, log(1+x) transform, selection of 2,000 highly variable genes, and subsampling to no more than 3,000 cells. Cluster labels are obtained via Leiden clustering (Traag et al., 2019) (resolution = 1.0) on PCA → UMAP neighbours computed with Scanpy (Wolf et al., 2018), providing a consistent unsupervised reference partition across all 53 datasets. This partition is a proxy benchmark target, not biological ground truth. The metadata inventory identifies three datasets with curated cell-type or lineage annotations (PanSci-Muscle Lineage, GSE130148-Lung celltype, and PanSci-T-cell Immune_subtype), three additional datasets with proxy trajectory or cluster annotations, and 47 datasets without curated labels in the available manifests. Curated-label results are, therefore, reported as a limited anti-circularity sensitivity analysis rather than as a universal validation of all 53 datasets. The random seed is fixed at 42 for reproducibility.

### 4.2 Evaluation metrics

We assessed latent representations along three axes comprising 20 metrics.

#### 4.2.1 Clustering quality (six metrics)

K -means clustering ( k matching the number of Leiden clusters) is applied to the latent space, and the predicted assignments are compared against the Leiden reference partition. Normalised mutual information (NMI) and adjusted Rand index (ARI) measure partition agreement. Average silhouette width (ASW ↑ ) quantifies intra- versus inter-cluster distance. The Davies–Bouldin index (DAV ↓ ) penalises cluster overlap. The Calinski–Harabasz score (CAL ↑ ) rewards compact, well-separated clusters. Mean absolute inter-dimensional Pearson correlation (COR) captures redundancy among latent dimensions.

#### 4.2.2 Dimensionality reduction evaluation (eight metrics)

We evaluated neighbourhood preservation between the latent space and its UMAP and t-SNE projections using a rank-based co-ranking framework (Lee and Verleysen, 2009). Let $Q_{ij}$ denote the co-ranking matrix comparing neighbour ranks in the latent space and projection. We compute the cumulative neighbourhood agreement curve $Q_{NX}\left(K\right)$ from $Q_{ij}$ , choose the local–global boundary at the maximum of the local-continuity meta-criterion $Q_{NX}\left(K\right)-K/N$ , and report the mean $Q_{NX}$ before that boundary as $Q_{\text{local}}$ and the mean after it as $Q_{\text{global}}$ . For each projection, we also report distance correlation (rank-distance agreement) and an overall quality score averaging distance correlation, $Q_{\text{local}}$ , and $Q_{\text{global}}$ .

#### 4.2.3 Latent space evaluation (six metrics)

We assessed the intrinsic geometry of the latent space via manifold dimensionality (intrinsic dimension estimate via PCA eigenvalue spectrum), spectral decay rate (slope of the sorted eigenvalue curve), participation ratio (effective number of active dimensions), anisotropy score (directional spread uniformity), noise resilience (stability under small Gaussian perturbations of the learned latent representation, not count-level dropout), and an overall quality score averaging the normalised component scores.

### 4.3 Statistical testing

#### 4.3.1 Per-comparison test

All method-versus-method comparisons use the two-sided Wilcoxon signed-rank test across the 53 datasets. The paired design (same dataset, different methods) removes between-dataset variance, and the non-parametric test is used because metric distributions across heterogeneous datasets are not guaranteed to be normal. Raw significance levels are denoted, where shown, as***p < 0.001, **p < 0.01, *p < 0.05, and ^ns^ not significant. In compact benchmark summary tables, boldface marks the best displayed mean. Full metric-level Wilcoxon/BH results are reported in the accompanying statistical tables rather than encoded as row-level significance markers.

#### 4.3.2 Multiple-comparison correction

Each results table constitutes a family of tests (baseline methods −1 ): × (metrics reported in that table), typically 30 to 100 hypotheses. Within each table we therefore apply the Benjamini–Hochberg FDR procedure at q=0.05 over the family and flag a comparison as “significant gain” only when it remains below the FDR threshold. For benchmark comparisons, BH-corrected p -values are evaluated at the metric level. The compact tables therefore avoid row-level significance symbols when a row contains a mixture of significant and non-significant metrics. Uncorrected raw p -values and BH-adjusted summaries are released with the code archive for readers who prefer a different correction.

#### 4.3.3 Matching

All baselines use the same preprocessing pipeline (Scanpy filter/HVG/normalisation; identical 15-NN graph), the same train–validation split, the same 200-epoch training budget with patience 30, and default hyperparameters as published by each method. Deep-learning baselines that expose a latent-dimension parameter are set to d=10 to match GAHIB; classical DR methods use their standard defaults. No per-method hyperparameter search was performed.

#### 4.3.4 Independence caveat

The 53 datasets come from distinct GEO/Zenodo/Figshare accessions, different tissues, and different primary publications, so they are plausibly independent at the dataset level. We do not claim strict independence: three pairs of datasets share a parent study (e.g., bccHm/sccHm), and some datasets are from the same protocol family. Wilcoxon results are therefore best interpreted as “aggregate evidence over a diverse cohort,” not as a strict i.i.d. hypothesis test.

### 4.4 Training configuration

All models are trained for 200 epochs with patience =30 (early stopping), hidden dimension =128 , latent dimension =10 , IB dimension =2 , learning rate $=10^{-4}$ , batch size =128 , and an NB likelihood. GAHIB-specific weights: β=0.1 , $\lambda_{\text{ib}}=0.5$ , $\lambda_{\text{hyp}}=5.0$ , with a GAT encoder on a 15-NN graph and 10×512 -node subgraph sampling.

## 5 Results

### 5.1 Results roadmap and evidence hierarchy

The results are presented in the order in which the claims should be read. Curated label and marker evidence are the primary basis for biological interpretation; the 53-dataset Leiden-proxy benchmark is a method-characterisation tier; robustness, sensitivity, and cost analyses define practical deployment boundaries. We retain the original main-figure sequence for auditability and to avoid removing any submitted visual evidence, but the narrative below consolidates the benchmark breadth into the claims that are directly supported. This ordering is important because the ARI/NMI reference used throughout the broad benchmark is a consistent Leiden partition, not a definitive biological annotation. Accordingly, benchmark gains are described as proxy-label performance, while statements about biological structure are restricted to curated/source-label systems, marker enrichment, pseudotime anchors, or explicitly labelled limitations.

#### 5.1.1 Reader-facing evidence map

The revised main manuscript carries the evidence needed for review in the main text, tables, and figure set within the same article package. Complete benchmark means, curated-label checks, marker-enrichment summaries, sensitivity sweeps, the dropout pilot, cost analysis, and implementation limits are reported in the relevant sections below. Source tables and figure-generation materials remain preserved in the repository/release materials for audit, but no biological or performance claim depends on an external-only table or figure.

### 5.2 Component ablation

We ablate GAHIB by progressively removing components, yielding five variants: Base VAE (MLP, no IB, no geometry), VAE + IB, VAE + Hyp, VAE + IB + Hyp, and the full GAHIB (GAT + IB + Hyp).

**FIGURE 4:**
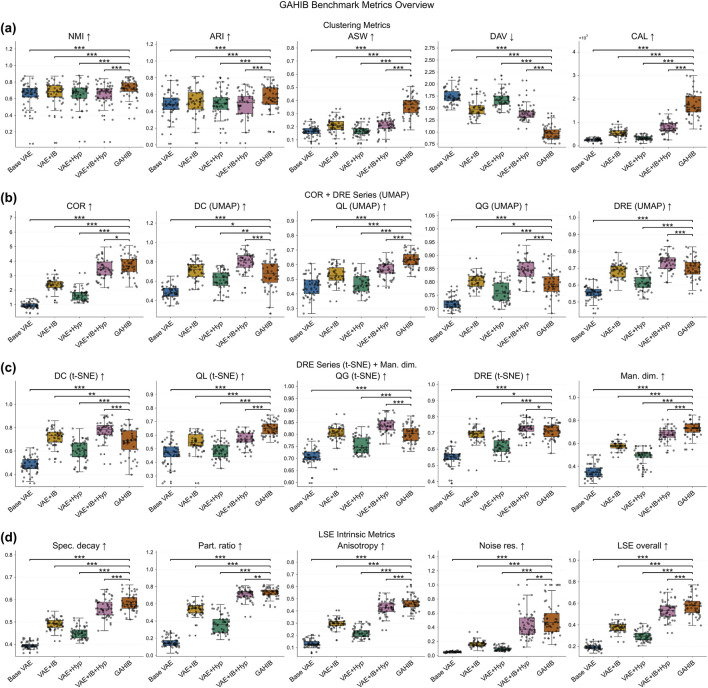
Component ablation across all 20 evaluation metrics spanning clustering, dimensionality reduction, and latent structure families. Boxes show the interquartile range over 53 datasets, and brackets denote Wilcoxon tests versus GAHIB. **(a)** clustering metrics (NMI, ARI, ASW, DAV, CAL); **(b)** UMAP-based projection-quality metrics (COR, DC, QL, QG, DRE); **(c)** t-SNE-based projection-quality metrics (DC, QL, QG, DRE, manifold dimensionality); **(d)** latent-structure / intrinsic LSE metrics (spectral decay, participation ratio, anisotropy, noise resistance, overall LSE).

Table 1 reports mean ± std across 53 datasets with Wilcoxon significance against GAHIB. Figure 4 shows boxplots across all 20 metrics.

**TABLE 1:** Component ablation: mean ± std across 53 datasets. Entries are compared with GAHIB using metric-level Wilcoxon signed-rank tests; DAV ↓ indicates that lower values are better, and boldface marks are the best displayed mean for each metric.

Method	NMI ↑	ARI ↑	ASW ↑	DAV ↓	DRE ↑	LSE ↑
Base VAE	0.651±.141	0.485±.152	0.165±.038	1.741±.160	0.553±.039	0.194±.025
VAE + IB	0.665±.137	0.512±.154	0.209±.051	1.489±.185	0.683±.042	0.378±.041
VAE + Hyp	0.653±.140	0.493±.153	0.168±.041	1.679±.168	0.615±.048	0.297±.048
VAE + IB + Hyp	0.650±.133	0.486±.150	0.215±.048	1.402±.166	0.735±.051	0.534±.081
**GAHIB**	0.722±.098	0.564±.142	0.364±.081	0.977±.139	0.699±.059	0.576±.086

#### 5.2.1 Information bottleneck (+IB)

Adding IB to the Base VAE improves ARI by + 0.027, ASW by + 0.044, and reduces DAV by − 0.252: the 2D bottleneck discards technical noise and keeps the most discriminative gene-expression patterns, which tightens cluster separation (higher ASW, lower DAV). The IB also supplies the structured 2D manifold coordinate $z^{\prime }$ required as an anchor for the hyperbolic geometry loss in the subsequent variants.

#### 5.2.2 Hyperbolic geometry (+Hyp)

Adding Lorentz geometry alone (VAE + Hyp) yields only marginal clustering improvement (NMI + 0.002) but substantially boosts DRE (0.553→0.615) and LSE (0.194→0.297) , indicating that the hyperbolic loss regularises the *global structure* of the latent manifold even when cluster separation is unchanged. *Hyp without IB is ineffective for clustering*: without a trained manifold coordinate $z^{\prime }$ , the Lorentz distance is computed against an untrained target and degenerates.

#### 5.2.3 Combined IB + Hyp

VAE + IB + Hyp achieves the best DRE (0.735) and LSE (0.534) among all MLP variants, confirming the synergy: IB provides the geometric anchor that enables the hyperbolic loss to structure the full latent space, not merely the 2D projection. The + 0.052 DRE gain over VAE + IB alone reflects improved global neighbourhood preservation—a direct benefit of the Lorentz geodesic penalty.

#### 5.2.4 Full GAHIB (GAT + IB + Hyp)

Replacing the MLP encoder with GAT yields the largest single improvement: NMI + 0.072, ARI + 0.078, ASW + 0.149, DAV − 0.425—all significant at p<0.001 . The GAT encoder aggregates information from cell-type-homophilic neighbours (mean attention homophily 0.812), producing pre-clustered representations that make the IB and hyperbolic losses markedly more effective. The GAT encoder, therefore, carries most of the clustering gain, while the geometric losses refine the resulting latent geometry.

### 5.3 Deep learning benchmark

We compared GAHIB against seven published deep learning methods: scVI (Lopez et al., 2018), CellBLAST (Cao et al., 2020), CLEAR (Han et al., 2022), SCALEX (Xiong et al., 2022), scDeepCluster (Tian et al., 2019), scDHMap (Tian T. et al., 2023), and scGNN (Wang et al., 2021). To address the graph-attention lineage raised during review, we also added three transparent PyTorch control implementations, displayed as “scGAC,” “SCEA,” and “scVAG.” These controls follow the design motifs of scGAC, SCEA, and scVAG (PCA/MLP/VAE front ends with graph-attention autoencoding on the same k NN graph family), but they are explicitly reported as transparent controls rather than exact upstream-code reproductions. All displayed methods are evaluated on the same 53 preprocessed datasets, use the same metric implementation, and use the same 200-epoch benchmark budget where training is applicable. The three control baselines additionally use k=15 , latent dimension 10, Adam, and full-batch graph training to match the GAHIB graph-construction policy as closely as possible.

**FIGURE 5:**
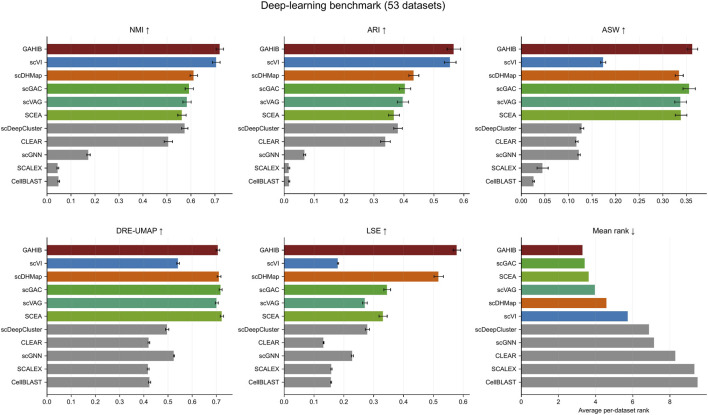
Deep learning and graph-attention control benchmark summary over 53 datasets. Bars show mean values with standard-error intervals for the six displayed summary metrics; lower values are better only for DAV and the average-rank panel. The green rows highlight the scGAC, SCEA, and scVAG controls, which are comparable PyTorch implementations of the reviewer-requested graph-attention design space rather than exact upstream-code reproductions. The figure supports the same balanced reading as Table 2: GAHIB leads in NMI, ARI, ASW, and LSE, whereas graph-attention controls remain competitive in DAV/DRE.

Table 2 reports the six principal display metrics for the seven published methods, the three graph-attention controls, and GAHIB. The expanded comparison changes the interpretation from a simple “first on all columns” statement to a more useful multi-objective profile: GAHIB has the highest mean NMI, ARI, ASW, and LSE among the displayed methods, whereas the scGAC control has the lowest DAV and the SCEA control has the highest DRE. scVI remains close to GAHIB on NMI (0.706 vs. 0.720) and is statistically indistinguishable on ARI in the paired signed-rank test (p=0.799) . scDHMap and the three graph-attention controls are also competitive on graph/projection metrics, including non-significant DRE comparisons for scDHMap (p=0.771) , the scGAC control (p=0.145) , and the scVAG control (p=0.855) . We therefore frame GAHIB as a balanced representation learner with strong clustering and latent-geometry performance, not as uniformly superior for every metric family or every biological use case (Figure 5).

**TABLE 2:** Deep learning and graph-attention control benchmark: mean ± std across 53 datasets. DAV ↓ indicates that lower values are better; bold marks the best displayed mean for each metric. The scGAC, SCEA, and scVAG rows are transparent PyTorch control implementations added for the graph-attention design-space comparison and should not be read as exact upstream-code reproductions.

Method	NMI ↑	ARI ↑	ASW ↑	DAV ↓	DRE ↑	LSE ↑
scVI	0.706±.116	0.555±.141	0.174±.039	1.699±.162	0.542±.037	0.180±.015
CellBLAST	0.049±.023	0.017±.014	0.026±.014	1.993±.174	0.424±.032	0.157±.014
CLEAR	0.506±.126	0.338±.119	0.118±.021	1.917±.108	0.421±.024	0.132±.009
SCALEX	0.045±.027	0.016±.022	0.045±.086	1.998±.171	0.418±.028	0.158±.014
scDeepCluster	0.574±.096	0.380±.104	0.129±.028	1.813±.140	0.497±.051	0.279±.051
scDHMap	0.612±.111	0.433±.122	0.335±.061	1.032±.153	0.711±.057	0.518±.114
scGNN	0.173±.049	0.068±.026	0.122±.018	1.725±.110	0.525±.019	0.229±.027
scGAC	0.593±.124	0.404±.139	0.356±.096	0.903±.156	0.718±.046	0.345±.080
SCEA	0.563±.126	0.367±.133	0.339±.089	0.923±.189	0.723±.049	0.332±.098
scVAG	0.584±.124	0.397±.136	0.338±.089	0.931±.154	0.701±.045	0.270±.059
GAHIB	0.720±.111	0.567±.162	0.363±.082	0.988±.156	0.707±.054	0.579±.089

### 5.4 Classical dimensionality reduction benchmark

We compare GAHIB with PCA, ICA, NMF, Truncated SVD, and DiffusionMap, each extracting 10 components from the same preprocessed data.

**FIGURE 6:**
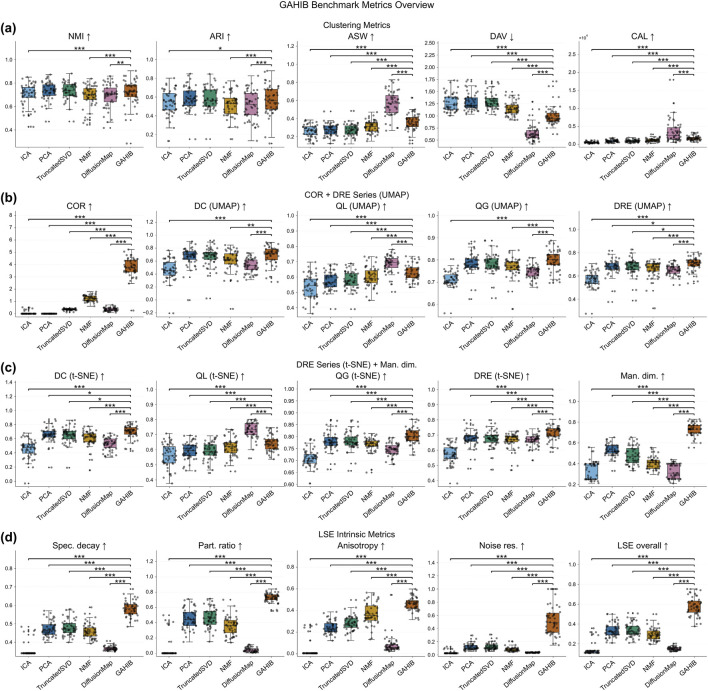
Classical dimensionality-reduction benchmark across 20 metrics for 6 methods. GAHIB leads the DRE and LSE families, while PCA and truncated SVD remain competitive on linearly separable datasets. **(a)** clustering metrics (NMI, ARI, ASW, DAV, CAL); **(b)** UMAP-based projection-quality metrics (COR, DC, QL, QG, DRE); **(c)** t-SNE-based projection-quality metrics (DC, QL, QG, DRE, manifold dimensionality); **(d)** latent-structure / intrinsic LSE metrics (spectral decay, participation ratio, anisotropy, noise resistance, overall LSE).

Table 3 shows that GAHIB achieves the best DRE (0.707) and LSE (0.576) means among the classical dimensionality-reduction methods. LSE differences are significant at p<0.001 versus all classical methods. DRE differences are also favourable but weaker against PCA (p=0.015) and Truncated SVD (p=0.027) , so the DRE result is interpreted as a best-mean advantage rather than a uniform p<0.001 effect. PCA and Truncated SVD achieve the highest NMI (0.735, 0.732) and ARI (0.586, 0.584), reflecting strong performance on linearly separable data. DiffusionMap leads on ASW (0.563) and DAV (0.645), benefiting from its nonlinear manifold structure; however, its clustering quality is substantially lower (NMI 0.693). GAHIB provides a strong overall balance, especially on complex datasets where nonlinear structure matters (Figure 6).

**TABLE 3:** Classical dimensionality-reduction benchmark: mean ± std across 53 datasets. Entries are compared with GAHIB using metric-level Wilcoxon signed-rank tests. DAV ↓ indicates that lower values are better, and boldface marks the best displayed mean for each metric.

Method	NMI ↑	ARI ↑	ASW ↑	DAV ↓	DRE ↑	LSE ↑
PCA	0.735±.075	0.586±.122	0.275±.067	1.285±.168	0.676±.068	0.336±.069
ICA	0.697±.097	0.547±.131	0.261±.067	1.282±.184	0.571±.070	0.139±.060
NMF	0.691±.094	0.524±.134	0.310±.068	1.148±.140	0.659±.072	0.294±.061
Trunc. SVD	0.732±.072	0.584±.118	0.271±.065	1.299±.164	0.678±.069	0.339±.071
DiffusionMap	0.693±.097	0.508±.151	0.563±.136	0.645±.165	0.654±.043	0.150±.018
**GAHIB**	0.721±.106	0.570±.150	0.362±.089	0.991±.183	0.707±.058	0.576±.095

### 5.5 Geometric VAE benchmark

We compare GAHIB against five geometric-VAE control configurations implemented under the same evaluation pipeline: Euclidean, Poincaré, PGM, Learnable PGM (L-PGM), and Hyperbolic–Wasserstein (HW). These controls use MLP encoders and identify whether changing the latent prior alone can match the combined graph-attention, bottleneck, and Lorentz-geometry design.

**FIGURE 7:**
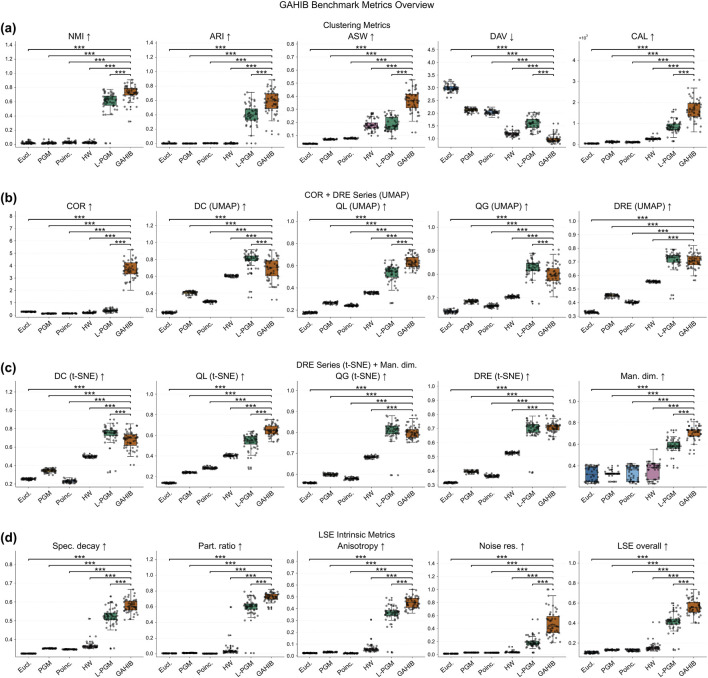
Geometric VAE benchmark across 20 metrics for 6 methods. Only the learnable PGM variant remains competitive; the other geometric priors collapse to weak embeddings, while GAHIB has the strongest reported means in this comparison. **(a)** clustering metrics (NMI, ARI, ASW, DAV, CAL); **(b)** UMAP-based projection-quality metrics (COR, DC, QL, QG, DRE); **(c)** t-SNE-based projection-quality metrics (DC, QL, QG, DRE, manifold dimensionality); **(d)** latent-structure / intrinsic LSE metrics (spectral decay, participation ratio, anisotropy, noise resistance, overall LSE).

Table 4 shows that GAHIB has the highest reported clustering, DAV, and LSE means in this controlled comparison, while Learnable PGM has a slightly higher DRE mean (0.715 vs. 0.707; non-significant against GAHIB in the paired test). Only the Learnable PGM variant approaches competitive clustering (NMI = 0.600); the remaining priors collapse to near-random partitions (NMI ≤ 0.027). A hyperbolic prior on its own is not enough: without neighbourhood-aware encoding and an anchored bottleneck, the geometry has no structure to regularise (Figure 7).

**TABLE 4:** Geometric VAE benchmark: mean ± std across 53 datasets. Entries are compared with GAHIB using metric-level Wilcoxon signed-rank tests. Boldface marks the best displayed mean for each metric.

Method	NMI ↑	ARI ↑	ASW ↑	DAV ↓	DRE ↑	LSE ↑
GM-VAE (Eucl.)	0.019±.013	0.001±.004	0.035±.001	2.984±.127	0.329±.006	0.105±.009
GM-VAE (Poinc.)	0.027±.013	0.003±.002	0.077±.003	2.036±.076	0.402±.005	0.127±.009
GM-VAE (PGM)	0.018±.011	0.000±.001	0.069±.003	2.126±.072	0.452±.009	0.131±.004
GM-VAE (L-PGM)	0.600±.147	0.404±.149	0.182±.054	1.608±.248	0.715±.066	0.415±.084
GM-VAE (HW)	0.025±.010	0.001±.004	0.181±.042	1.185±.083	0.555±.005	0.156±.041
**GAHIB**	0.726±.104	0.577±.154	0.365±.079	0.973±.143	0.707±.050	0.562±.078

### 5.6 Disentanglement regularisation comparison

We compare GAHIB against four disentanglement regularisation strategies applied to a base VAE with MLP encoder: β -VAE, DIP-VAE, β -TC-VAE, and InfoVAE.

**FIGURE 8:**
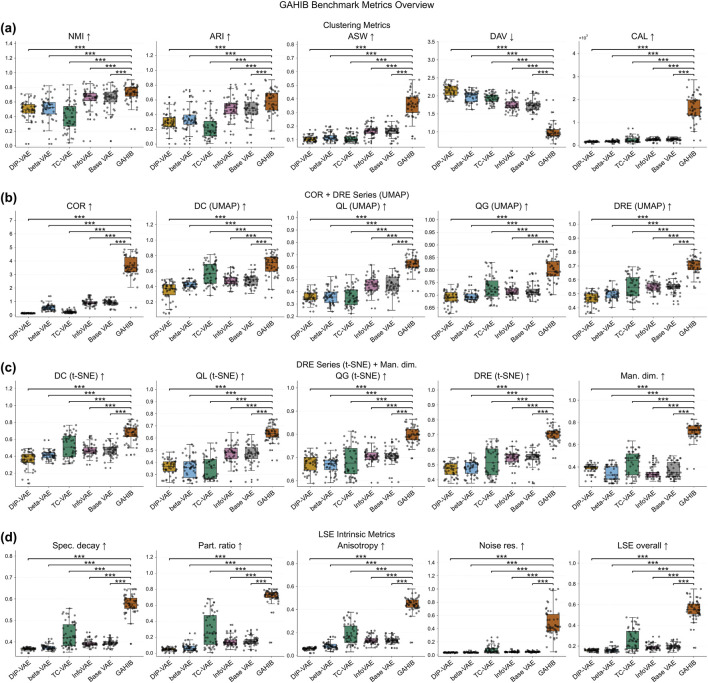
Disentanglement regularisation comparison across 20 metrics. Posterior regularisers reduce clustering quality relative to the base VAE, whereas GAHIB’s structural prior remains consistently stronger. **(a)** clustering metrics (NMI, ARI, ASW, DAV, CAL); **(b)** UMAP-based projection-quality metrics (COR, DC, QL, QG, DRE); **(c)** t-SNE-based projection-quality metrics (DC, QL, QG, DRE, manifold dimensionality); **(d)** latent-structure / intrinsic LSE metrics (spectral decay, participation ratio, anisotropy, noise resistance, overall LSE).

Table 5 shows that GAHIB has the highest reported means, with p<0.001 significance across the reported metrics. β -VAE, DIP-VAE, and TC-VAE *decrease* clustering quality below the unregularised Base VAE (TC-VAE NMI 0.404 vs. Base 0.647). These regularisers impose strong constraints on the posterior, encouraging independent or factorised latent dimensions. In scRNA-seq data, however, the dominant sources of variation are co-regulated gene programs, so forcing orthogonality disrupts the representation that the encoder would otherwise learn. InfoVAE, which relaxes the factorisation constraint, is nearly neutral (NMI 0.649 vs. Base 0.647), consistent with this interpretation. GAHIB achieves NMI 0.722 ( + 0.073 over InfoVAE, + 0.318 over TC-VAE), indicating that a *geometric* structural prior (IB + Hyp) is more effective than posterior regularisation for single-cell clustering (Figure 8).

**TABLE 5:** Disentanglement comparison: mean ± std across 53 datasets. Entries are compared with GAHIB using metric-level Wilcoxon signed-rank tests. Boldface marks the best displayed mean for each metric.

Method	NMI ↑	ARI ↑	ASW ↑	DAV ↓	DRE ↑	LSE ↑
Base VAE	0.647±.138	0.480±.149	0.164±.039	1.740±.155	0.548±.041	0.195±.026
β -VAE	0.513±.156	0.337±.151	0.119±.030	1.980±.155	0.495±.036	0.161±.020
DIP-VAE	0.491±.122	0.306±.116	0.105±.021	2.142±.160	0.467±.047	0.159±.010
TC-VAE	0.404±.193	0.234±.160	0.109±.030	1.936±.131	0.551±.084	0.263±.102
InfoVAE	0.649±.140	0.484±.151	0.164±.038	1.754±.166	0.551±.040	0.190±.027
**GAHIB**	0.722±.113	0.576±.161	0.356±.086	0.994±.192	0.700±.058	0.559±.093

### 5.7 Encoder architecture comparison

To isolate the effect of the encoder, we fix the GAHIB loss configuration (IB + Hyp) and vary only the encoder—MLP, Transformer (multi-head self-attention), and GAT.

**FIGURE 9:**
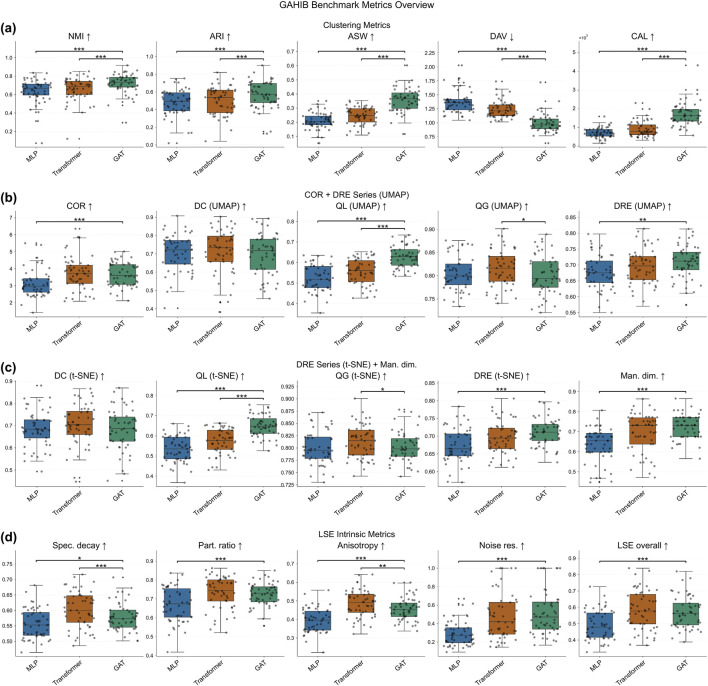
Encoder architecture comparison across 20 metrics with the IB + Hyp objective fixed. GAT improves the clustering metrics and DAV under this benchmark setting, while DRE/LSE remain close to the transformer control. **(a)** clustering metrics (NMI, ARI, ASW, DAV, CAL); **(b)** UMAP-based projection-quality metrics (COR, DC, QL, QG, DRE); **(c)** t-SNE-based projection-quality metrics (DC, QL, QG, DRE, manifold dimensionality); **(d)** latent-structure / intrinsic LSE metrics (spectral decay, participation ratio, anisotropy, noise resistance, overall LSE).

Table 6 shows a clear hierarchy: GAT > Transformer > MLP across all clustering metrics, all significant at p<0.001 . GAT achieves NMI 0.723 versus Transformer 0.659 and MLP 0.640. The GAT encoder benefits from explicit graph structure (15-NN graph), while the Transformer’s self-attention provides partial neighbourhood awareness. This confirms that graph-structured encoding is a stronger inductive bias than self-attention alone (Figure 9).

**TABLE 6:** Encoder comparison: mean ± std across 53 datasets. Entries are compared with the GAT encoder using metric-level Wilcoxon signed-rank tests. Boldface marks the best displayed mean for each metric.

Encoder	NMI ↑	ARI ↑	ASW ↑	DAV ↓	DRE ↑	LSE ↑
MLP	0.640±.132	0.479±.153	0.210±.057	1.370±.193	0.677±.054	0.490±.091
Transformer	0.659±.129	0.504±.158	0.246±.059	1.238±.150	0.695±.056	0.583±.121
**GAT**	0.723±.107	0.571±.152	0.359±.083	0.993±.170	0.709±.052	0.570±.097

### 5.8 Graph convolution operator sweep

We compare six graph convolution operators within the GAHIB framework: GCN, GraphSAGE, Chebyshev (Cheb), TAG, GraphTransformer, and GAT.

**FIGURE 10:**
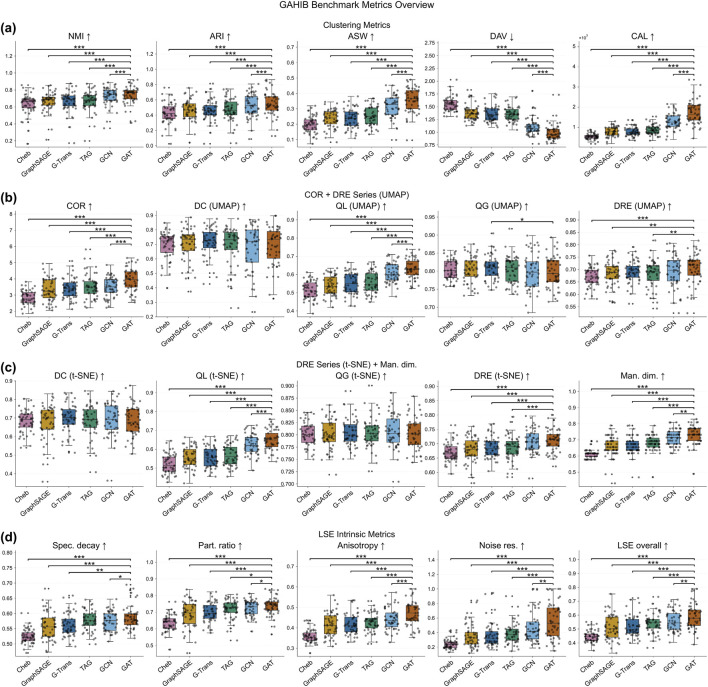
Graph convolution operator sweep across 20 metrics within the GAHIB framework. GAT is the strongest overall operator in the displayed summary metrics, including DRE, while GCN and GraphTransformer remain close on geometry-oriented scores. **(a)** clustering metrics (NMI, ARI, ASW, DAV, CAL); **(b)** UMAP-based projection-quality metrics (COR, DC, QL, QG, DRE); **(c)** t-SNE-based projection-quality metrics (DC, QL, QG, DRE, manifold dimensionality); **(d)** latent-structure / intrinsic LSE metrics (spectral decay, participation ratio, anisotropy, noise resistance, overall LSE).

Table 7 shows GAT as the strongest operator overall. It achieves the best NMI (0.744), ARI (0.551), ASW (0.358), DAV (0.990), and LSE (0.591), while GCN offers a competitive alternative (NMI 0.727). The attention mechanism in GAT provides learnable, cell-specific aggregation weights, improving cluster separation beyond fixed-weight message passing (Figure 10).

**TABLE 7:** Graph convolution sweep: mean ± std across 53 datasets. Entries are compared with GAT using metric-level Wilcoxon signed-rank tests. Boldface marks the best displayed mean for each metric.

Conv. Type	NMI ↑	ARI ↑	ASW ↑	DAV ↓	DRE ↑	LSE ↑
GCN	0.727±.104	0.522±.150	0.314±.073	1.102±.167	0.693±.065	0.558±.074
GraphSAGE	0.666±.113	0.470±.133	0.236±.058	1.379±.147	0.682±.049	0.511±.088
Cheb	0.633±.120	0.428±.139	0.197±.054	1.548±.143	0.670±.039	0.445±.040
TAG	0.671±.115	0.474±.142	0.252±.060	1.353±.139	0.684±.054	0.540±.069
GraphTransformer	0.661±.120	0.460±.138	0.237±.063	1.361±.160	0.692±.043	0.519±.076
**GAT**	0.744±.100	0.551±.152	0.358±.079	0.990±.158	0.705±.059	0.591±.091

## 6 Biological interpretation

We performed *post hoc* analysis of GAHIB’s trained representations across all 53 datasets. All results use the same trained models as the benchmark experiments. This section separates primary biological evidence from intermediate representation diagnostics. The broad all-dataset figures are useful for checking whether the trained models behave consistently, but they do not by themselves establish biological discovery because many datasets have only Leiden proxy labels. Biological claims therefore rely on the curated-label and marker evidence summarised below, with the all-dataset visualizations retained as the original complete evidence record.

### 6.1 Curated-label case-study evidence and limitations

Three evaluation datasets carry multi-level curated biological labels: PanSci-Muscle (Lineage, 7 levels), Lung-Dev (cell type, 13 levels), and PanSci-T-cell (immune subtype, 18 levels). These datasets provide the available non-Leiden checks. The results are deliberately mixed. In PanSci-Muscle, GAHIB supports a lineage-oriented view: the curated-label analysis shows higher curated ASW and slightly higher ARI than scVI in the compact pilot, and the marker-enrichment analysis identifies significant endothelial, stromal, and immune marker recovery within GAHIB-aligned clusters (Tables 8, 9). The same marker-enrichment screen did not yield Benjamini–Hochberg significant rows for the Lung-Dev or PanSci-T-cell panels, so the marker table is deliberately limited to the Muscle atlas rather than promoted as a universal marker-recovery result. In PanSci-T-cell, scVI better matches the fine immune-subtype taxonomy, so we use this system as a contrast rather than as evidence of context-independent GAHIB advantage. Lung-Dev is intermediate: GAHIB has higher curated ASW, whereas scVI has higher curated NMI/ARI. We, therefore, frame GAHIB’s biological utility as context-specific: it is strongest when hierarchy or lineage organisation is the desired signal, and it is complementary to Euclidean VAEs for fine subtype taxonomies.

**TABLE 8:** Curated-label evaluation on the three annotated systems used as non-Leiden sensitivity checks. Values summarise the compact pilot; the table is interpreted as context-specific evidence rather than a universal biological-superiority claim.

​	​	GAHIB			scVI		
Dataset	Curated label	NMI	ARI	ASW	NMI	ARI	ASW
PanSci-muscle	Lineage (7)	0.645	0.549	**0.396**	**0.657**	0.522	0.210
Lung-Dev	Celltype (13)	0.615	0.438	**0.325**	**0.700**	**0.575**	0.231
PanSci-T-cell	Immune_subtype (18)	0.253	0.140	0.094	**0.493**	**0.349**	**0.095**

Bold values indicate the best performance for each metric.

**TABLE 9:** GAHIB-cluster marker-enrichment hypergeometric tests reaching Benjamini–Hochberg significance (q<0.05) in the compact marker panel. The significant rows are limited to the Muscle atlas and support a lineage-oriented interpretation of that case study.

Dataset	Curated match	|markers| in universe	Overlap	Expected	p	$q_{\text{BH}}$
Muscle	Endothelial	3	2	0.05	$6.5\times 10^{-4}$	<0.05
Muscle	Stromal	5	2	0.08	$2.1\times 10^{-3}$	<0.05
Muscle	Immune	2	2	0.03	$2.2\times 10^{-4}$	<0.05

### 6.2 Primary analysis families

The interpretation is organised into four families. First, curated/source-label and marker analyses provide non-Leiden biological validation (Section 6.1). Second, hyperbolic hierarchy and pseudotime analyses test whether Lorentz norm tracks differentiation-like structure (Section 6.7). Third, robustness and sensitivity analyses separate completed checks, such as multi-seed and k -sensitivity summaries, from the bounded count-level dropout pilot (Sections 7, 7.4). Fourth, computational-cost analyses quantify deployability under the tested subsampling regime (Section 7.2). Embedding maps, bottleneck plots, and all-dataset gene-attribution grids are treated as diagnostics that support these four families, not as independent proof of biological innovation.

### 6.3 Sensitivity and source-label validation

**FIGURE 11:**
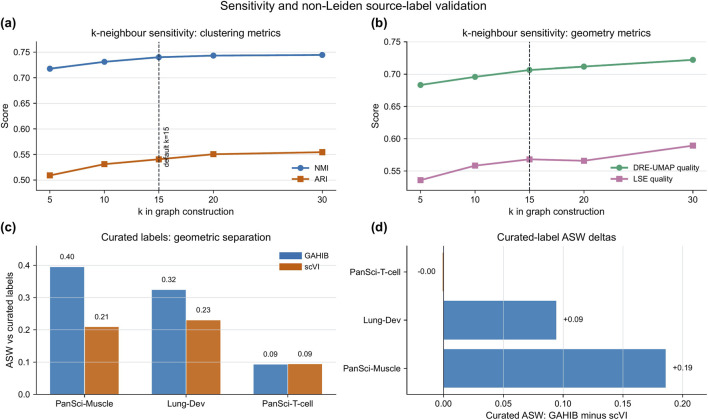
Sensitivity and non-Leiden source-label validation. **(a)** Clustering metrics across k -nearest-neighbour graph sizes show that the default k=15 is an interior, stable choice. **(b)** Geometry-oriented DRE-UMAP and LSE scores remain comparable across the same k sweep. **(c)** Curated-label ASW compares the geometry of GAHIB and scVI on all three annotated systems. **(d)** Summary of the ASW delta (GAHIB minus scVI) for the same three systems, including the near-zero T-cell endpoint. Full NMI/ARI/ASW values remain reported in Table 8, including endpoints where scVI is higher.

Figure 11 provides two bounded validation checks: sensitivity of the graph neighbourhood size and comparison against available curated/source labels. Panels (a) and (b) show that the default k=15 lies in the interior of a stable neighbourhood sweep rather than at an isolated optimum. Panels (c) and (d) show the curated/source-label ASW comparison for all three annotated systems. Table 8 retains the full NMI/ARI/ASW comparison against scVI. This split keeps the label-matching result visible: scVI is higher for several discrete NMI/ARI source-label partitions, especially the fine PanSci-T-cell immune-subtype taxonomy, whereas GAHIB is stronger for the PanSci-Muscle ARI endpoint and for curated-label ASW in the compact Muscle and Lung pilots; the T-cell ASW endpoint is essentially tied and is marginally higher for scVI. These source-label comparisons reduce the circularity of Leiden-only evaluation where curated annotations exist, but they remain a limited sensitivity analysis rather than a claim of universal biological superiority across all 53 datasets.

### 6.4 Information bottleneck structure

**FIGURE 12:**
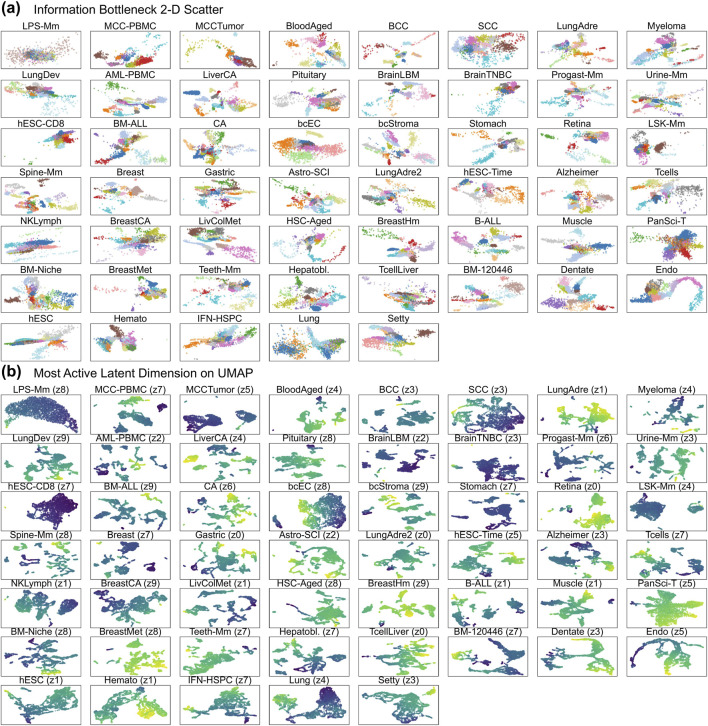
Information bottleneck structure analysis. **(a)** 2D IB scatter of the compressed manifold coordinate $z^{\prime }\in R^{2}$ , coloured by Leiden cluster, showing that cluster structure is retained after 10D→2D compression. **(b)** UMAP coloured by the most active latent dimension (highest variance), indicating that individual dimensions capture distinct biological axes.

Figure 12 visualises the IB compression. Panel (a) shows 2D IB scatter coloured by cell type, demonstrating that the bottleneck preserves cluster structure even at extreme compression (10D→2D) . Panel (b) overlays the most active latent dimension on UMAP, showing that different dimensions capture distinct biological axes. All 10 latent dimensions are active across all 53 datasets (Table 10), with no dimension collapse.

**TABLE 10:** Per-dataset interpretation summary. Twelve representative datasets are shown; the table mean is, therefore, computed over these 12 rows, while the full-cohort mean $\left(\rho_{s}=0.329\right)$ reported in the text is computed over all 53 datasets. Hom. indicates GAT attention homophily; ${\bar{r}}_{L}$ is the mean Lorentz norm; $\rho_{s}$ is the stemness–norm Spearman correlation. Significance stars in the corresponding column indicate the per-dataset Wilcoxon test (***p < 0.001, *p < 0.05); MO is the marker overlap fraction; GO denotes the number of significantly enriched GO terms.

Dataset	Hom	${\bar{r}}_{L}$	$\rho_{s}$	MO	GO
TcellLiver	0.845	4.14	$0.120^{***}$	0.426	46
MCCTumor	0.861	1.17	$0.501^{***}$	0.460	50
bcEC	0.798	1.66	$-0.099^{***}$	0.358	33
CA	0.808	1.89	$0.505^{***}$	0.255	23
Hepatobl	0.869	1.81	$0.275^{***}$	0.218	31
Breast	0.851	1.84	$0.615^{***}$	0.315	0
Endo	0.878	1.66	$0.386^{***}$	0.350	0
Pituitary	0.789	2.31	$0.043^{*}$	0.193	30
Setty	0.860	1.46	$0.179^{***}$	0.407	21
hESC	0.758	1.73	$0.454^{***}$	0.363	41
Lung	0.827	1.37	$0.274^{***}$	0.295	21
Dentate	0.874	1.71	$0.673^{***}$	0.402	0
**Mean (12)**	**0.835**	**1.90**	**0.327**	**0.337**	**24.7**

Bold values indicate the best performance for each metric.

### 6.5 Hyperbolic hierarchy

**FIGURE 13:**
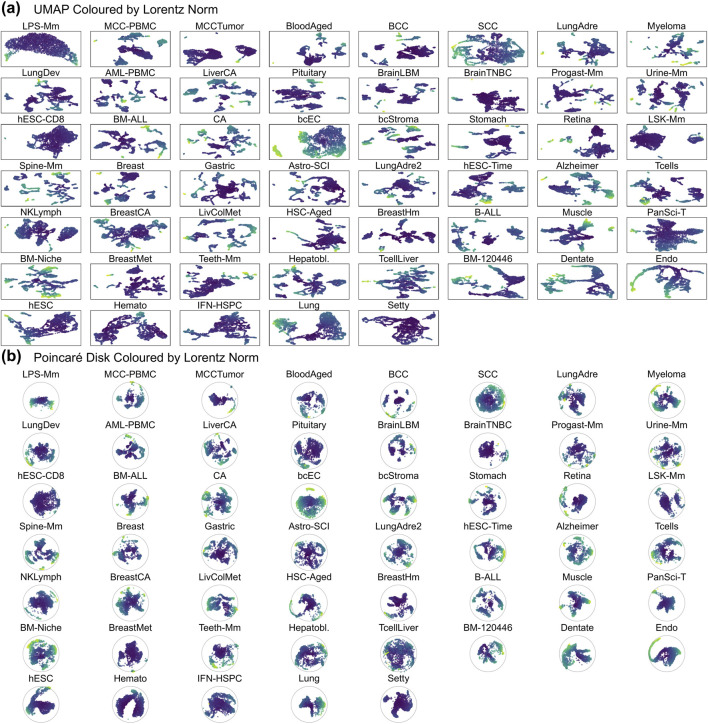
Hyperbolic hierarchy analysis. **(a)** UMAP embeddings coloured by Lorentz norm $\|\text{ExpMap}\left(z_{i}\right){\|}_{L}$ . Purple indicates low norm (stem/progenitor), and yellow indicates high norm (differentiated). **(b)** Poincaré disk projections coloured by the same norm, showing the radial gradient from origin to boundary that encodes developmental hierarchy.

Figure 13 colours cells by their Lorentz norm on UMAP (Panel (a)) and Poincaré disk (Panel (b)). Stem and progenitor cells have lower norms (closer to the origin), whereas differentiated cells have higher norms (closer to the boundary).

This is quantified by the stemness–norm Spearman correlation $\rho_{s}$ in Table 10, which is positive in 45/53 datasets (mean $\rho_{s}=0.329$ ) and reaches significance in the majority; the remaining eight datasets show small-magnitude negative correlations, consistent with non-developmental tissue composition. The strongest correlations appear in developmental datasets: dentate $\left(\rho_{s}=0.673\right)$ , breast cancer (0.615), MCCTumor (0.501), and colorectal adenocarcinoma (0.505).

### 6.6 Gene ontology biological process enrichment

To further confirm that individual GAHIB latent dimensions encode coherent biological programmes, we performed GO biological process enrichment analysis on four representative datasets (dentate gyrus, hematopoiesis, Setty hematopoietic, and hESC) covering neural, haematopoietic, and embryonic lineages. For each latent dimension $L_{k}$ , we computed the Pearson correlation between $L_{k}$ and every gene across cells, selected the top 100 correlated genes, and queried them against a curated hallmark-style panel of biological process gene sets using a hypergeometric test. Significance used Benjamini–Hochberg adjusted p<0.05 .

**FIGURE 14:**
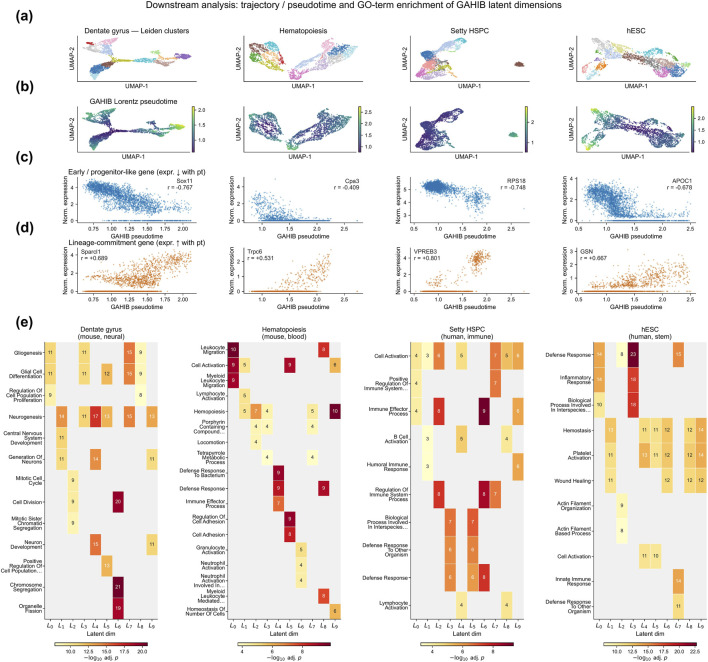
Downstream analysis of GAHIB. Panels **(a)**–**(d)** support GAHIB’s Lorentz-norm pseudotime from the original gene expression on four datasets (dentate gyrus, haematopoiesis, Setty HSPC, and hESC). **(a)** 2D UMAP coloured by Leiden clusters. **(b)** Same embedding coloured by GAHIB pseudotime (viridis: dark → stem, light → differentiated). **(c)** Scatter of GAHIB pseudotime versus the top negatively correlated HVG (progenitor-like; expression decreases with $r_{i}$ ), with the Pearson r annotated. **(d)** Scatter of GAHIB pseudotime versus the top positively correlated HVG (lineage-commitment; expression increases with $r_{i}$ ). **(e)** Tissue-specific GO biological process enrichment of the 10 latent dimensions for four independently trained GAHIB models; each heatmap column is one latent dimension, and each row is an enriched term (cell colour $=-\log_{10}$ adjusted p -value). Within these datasets, latent dimensions highlight tissue-appropriate programmes without reusing the same terms across tissue contexts.

Panel (e) of Figure 14 summarises the top-three enriched terms per latent dimension for each tissue context. GAHIB’s latent dimensions partition biological programs compartmentally: $L_{0}$ and $L_{3}$ capture immune-related pathways (inflammatory response, defense response; $-\log_{10}$ adj. p up to 22.7); $L_{1},L_{4},L_{5},L_{9}$ encode neural programmes (neurogenesis, neuron development, CNS development); $L_{2}$ and $L_{6}$ recover cell-cycle machinery (mitotic cell cycle and chromosome segregation); $L_{7}$ specifically captures glial biology (glial cell differentiation). Haemostasis and platelet activation are localised to $L_{8}$ and $L_{9}$ . The absence of off-diagonal enrichment within each functional category confirms that latent dimensions are functionally specialised rather than redundant, and the high significance levels (adj. $p\leq 10^{-9}$ throughout) confirm that the identified pathways are not noise.

#### 6.6.1 Tissue-context specificity

Because GAHIB is trained independently on each dataset, the latent dimensions learn tissue-specific biological programs rather than generic pathways. Panel (e) of Figure 14 displays the top-three enriched GO BP terms per latent dimension for each of the four datasets, trained as separate GAHIB models. The enrichment is context-dependent: **dentate gyrus** (mouse, neural) recovers gliogenesis, neurogenesis, central nervous system development, and glial cell differentiation (adj. p down to $10^{-21}$ ); **haematopoiesis** (mouse, blood) identifies leukocyte migration, hemopoiesis, granulocyte/neutrophil activation, and defence response to bacterium; **Setty HSPC** (human, immune) yields B-cell activation, immune effector process, and lymphocyte activation; and **hESC** (human, stem/haemostatic) captures haemostasis, platelet activation, wound healing, and inflammatory response (adj. p down to $10^{-23}$ ). Terms from one tissue context never appear in another—the model trained on brain tissue does not recover platelet biology, and the blood-trained model does not recover neurogenesis. This tissue specificity, discovered *without any supervision*, indicates that GAHIB learns disentangled, biologically meaningful programs that reflect the underlying cellular biology rather than generic transcriptional noise.

### 6.7 Pseudotime validation via gene expression

Rather than evaluating the Lorentz-norm pseudotime against an algorithmic reference (such as Scanpy’s diffusion pseudotime), we validate it directly from the original gene expression: a faithful developmental pseudotime should order cells so that known progenitor-like programs decrease along $r_{i}$ while lineage-commitment programs increase. Concretely, for each dataset we (i) train an independent GAHIB model, (ii) pick the cell with the smallest Lorentz norm as the developmental root, (iii) compute the Pearson correlation $r_{g}=corr\left(x_{g},r_{\cdot }\right)$ between every highly-variable gene $x_{g}$ and GAHIB’s pseudotime, and (iv) report the top negatively-correlated gene (early/progenitor-like) and the top positively-correlated gene (lineage-commitment) per dataset.

In Figure 14, Panels (a)–(d) summarise the gene-expression supporting analysis. Across these four datasets, the top negatively- and positively-correlated HVGs show well-separated expression trajectories along $r_{i}$ (Panels (c) and (d)), indicating that GAHIB’s hyperbolic pseudotime is consistent with dataset-specific gene programs rather than only a statistical artefact of the embedding.

### 6.8 Gene attribution and cross-dataset overview

GAHIB recovers 31.5% on average of DE marker genes within its top attributed genes (Table 10) and identifies 22 significant GO enrichment terms per dataset on average (34.3 among the 34 datasets with non-zero enrichment; 19 datasets produce no enriched terms at BH-adj. p<0.05 ).

**FIGURE 15:**
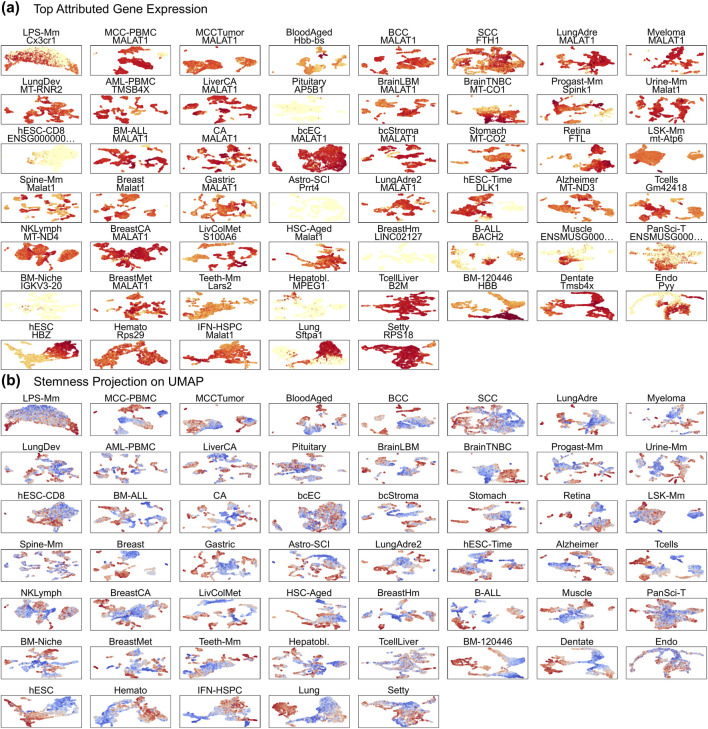
Gene attribution and stemness summary across all 53 datasets. **(a)** UMAP coloured by the top attributed gene (decoder Jacobian |∂g/∂z| ) per dataset; coherent spatial gradients indicate that GAHIB captures biologically meaningful gene programs. **(b)** Stemness score projected on UMAP (red = high stemness, blue = low), showing the stem → differentiated gradient recovered by the hyperbolic latent geometry.

Figure 15 provides an integrated overview combining top gene attribution (Panel (a)) with stemness projections (Panel (b)). Mean GAT attention homophily is 0.812, with 46/53 datasets above 0.75 (minimum 0.458 for lung), indicating that the attention mechanism captures biologically coherent cell–cell interactions in most but not all contexts.

Table 10 summarises key interpretation metrics per dataset.

## 7 Robustness, efficiency, and sensitivity

We provide four additional studies covering latent-dimension selection, reproducibility across random seeds, computational cost, and hyperparameter sensitivity. All studies reuse the 53 scRNA-seq datasets and the same 20-metric evaluation protocol.

### 7.1 Latent dimension ablation and multi-seed reproducibility

Supplementary Figure 1a sweeps the latent dimension d∈{3,5,10,20,50} while holding the IB dimension fixed at 2 and all other hyperparameters at their default values. Clustering quality peaks at d=3 (NMI = 0.652, ASW = 0.418) and degrades monotonically as d increases, with d=50 producing markedly lower ASW (0.221), consistent with distance-concentration risks in high-dimensional Euclidean space. We retain d=10 as the default because it preserves sufficient capacity for downstream tasks (trajectory inference and gene attribution) while remaining within the observed low-variation region.

Supplementary Figure 1b retrains GAHIB with five random seeds ({42, 123, 456, 789, 2024}) on each of the 53 datasets. Aggregate statistics are NMI = 0.739 ± 0.022, ARI = 0.546 ± 0.041, and ASW = 0.359 ± 0.020, indicating low seed-to-seed variation in this run set: the within-dataset standard deviation across seeds is typically less than 3% of the mean.

### 7.2 Computational cost and scaling

Supplementary Figure 2a compares training time and peak GPU memory for three architectures across the 53 datasets. GAHIB requires 22.0 ± 3.2 s per dataset and 0.11 GB peak memory—a 41% overhead over Base VAE (15.6 ± 2.4 s, 0.055 GB) and 10% over VAE + IB + Hyp (20.0 s, 0.059 GB). The additional cost comes primarily from subgraph sampling and GAT message passing.

Supplementary Figure 2b examines cell-count scaling on 10 representative datasets, each uniformly random-subsampled to n∈{500,1000,2000,3000} cells and retrained from scratch. Training time is flat (22.0–22.6 s across all four sizes) because the per-epoch cost is largely driven by a fixed number of subgraph training steps (10 subgraphs × 512 nodes), not the dataset size; the measurement, therefore, reports the steady-state cost rather than a true asymptotic.

Supplementary Figure 2c isolates the mini-batch-size effect by holding n fixed at 2,000 cells (uniform random subsample from five datasets) and sweeping the training batch size through {32,64,128,256,512} . The per-epoch wall-clock time is essentially constant (0.59–0.61 s/epoch, relative spread <4% ) across the whole range. This observed insensitivity is consistent with GAHIB’s subgraph-paced schedule: each epoch processes a fixed number of 512-node subgraphs regardless of the DataLoader batch, so the batch parameter controls only the downstream mini-batch reduction inside each subgraph. Practically, within the tested batch/subsampling settings, batch-size effects were small, and the default of 128 remained stable.

As a descriptive statistical check on the existing cost outputs, we also ran one-way ANOVA over the three architectures with complete paired records for 52 datasets. Training time differs by architecture ( $F_{2,102}=155.09$ , $p=1.17\times 10^{-31}$ ), as does peak memory ( $F_{2,102}=62057.39$ , $p=4.32\times 10^{-158}$ ), whereas the number of actual training epochs does not differ significantly ( $F_{2,102}=1.34$ , p=0.265 ). These tests are descriptive summaries of the measured benchmark outputs, not new asymptotic scaling claims.

### 7.3 Bounded count-level dropout pilot

The LSE noise-resilience score above uses Gaussian perturbations of the learned latent representation and should not be interpreted as scRNA-seq dropout robustness. To address this distinction, we ran a bounded pilot on the three annotated systems available for curated-label validation: PanSci-Muscle, Lung-Dev, and PanSci-T-cell. The pilot directly masked 0%, 20%, 40%, and 60% of non-zero count entries before fitting and evaluating GAHIB, scVI, and PCA under both curated/source labels and Leiden labels (maximum 2,000 cells, 20 epochs, seed 42).

The pilot is useful as a stress test, but it is not a full robustness benchmark. All methods degrade as masking increases, and the method ranking remains context-dependent: scVI is stronger on Lung and T-cell curated labels, PCA is competitive in several Leiden-label settings, and GAHIB does not show universal dropout resistance. We, therefore, use this analysis only to delimit the scope of the Gaussian-noise metric and state that count-level dropout robustness remains bounded to these named systems and settings.

### 7.4 Hyperparameter sensitivity

We swept each of four key hyperparameters, holding the others at default values: the KL weight β∈{0.01,0.05,0.1,0.5,1.0} , the IB weight $\lambda_{\text{ib}}\in \left\{0.1,0.25,0.5,1.0,2.0\right\}$ , the Lorentz weight $\lambda_{\text{hyp}}\in \left\{1.0,2.5,5.0,10.0,20.0\right\}$ , and the graph size k∈{5,10,15,20,30} . Supplementary Figure S3 shows bounded variation across all four tested sweeps.

-
β : NMI varies within a narrow range of [0.723,0.741] (total spread Δ=0.019 ).
-
$\lambda_{\text{ib}}$ : [0.733,0.745] (Δ=0.012) .
-
$\lambda_{\text{hyp}}$ : [0.711,0.755] (Δ=0.044) .
-
k : [0.718,0.744] (Δ=0.027) .

The Lorentz weight shows the largest effect: smaller $\lambda_{\text{hyp}}$ improves clustering (NMI = 0.755 at $\lambda_{\text{hyp}}=1$ ) but correspondingly reduces hierarchical structure in the latent space, suggesting a trade-off in these settings between cluster separation and hyperbolic geometry preservation. The k -sweep supports the default k=15 as a conservative midpoint: performance is stable from k=10 to 20, while very small graphs risk fragmenting local neighbourhoods, and very large graphs can blur rare-cell boundaries. The default $\lambda_{\text{hyp}}=5$ balances clustering and hierarchy objectives.

### 7.5 Companion reader site

The companion reader site provides a navigation layer for the public benchmark description and reproducibility handoff. Supplementary Figure S4 shows the current route map and is included as an open-science pointer; it is not used as quantitative evidence for model performance or as a source of additional biological claims.

## 8 Discussion

### 8.1 A balanced embedding for multi-objective single-cell analysis

GAHIB’s practical value is balance: in this benchmark it remains simultaneously strong across clustering, geometry-preservation, and latent-structure metrics. The benchmark suite shows that other methods perform best on different parts of the problem. scVI comes close to GAHIB on NMI and ARI in the deep-learning benchmark, scDHMap is competitive on geometry-aware metrics, and PCA or truncated SVD remain strong on datasets with mostly linear structures. In real single-cell workflows, however, users rarely train separate embeddings for clustering, visualisation, pseudotime, and interpretation; they want one representation that supports all of these downstream uses without manual switching between models.

GAHIB reduces that trade-off in the tested settings. Compared with scVI, it tends to preserve stronger cluster margins and latent geometry; compared with scDHMap, it improves separation while keeping the hierarchy signal; compared with classical methods, it is more reliable when tumour heterogeneity and developmental continua coexist in the same dataset. In settings where both neighbourhood fidelity and branching organisation matter, GAHIB is most appropriately interpreted as a balanced general-purpose embedding rather than a specialised hyperbolic model. This statement is restricted to the methods and proxy-label settings in the benchmark. The curated-label pilot shows that scVI is preferable for some fine immune-subtype tasks. The added scGAC, SCEA, and scVAG controls test the graph-attention design space under a common PyTorch benchmark policy, but exact upstream-code reproductions of those lineage methods should still be viewed as separate comparators rather than as methods that GAHIB has empirically surpassed here.

### 8.2 The GAT encoder explains much of the clustering gain

Across the architectural ablation, encoder, and graph-convolution studies, the GAT encoder consistently emerges as the single most impactful architectural component. The ablation (Section 5.2) reveals that replacing the MLP encoder with GAT (VAE + IB + Hyp → GAHIB) yields the largest performance gain on every clustering metric (NMI +0.072, ARI +0.078), surpassing the effect of adding either the information bottleneck or hyperbolic geometry individually. The encoder comparison (Section 5.7) further confirms a clear and statistically significant hierarchy: GAT > Transformer > MLP.

GAHIB’s gains on *cluster-separation* metrics—ASW (+0.149 vs. VAE + IB + Hyp), CAL, and DAV—are consistent with GAT-based neighbourhood aggregation. Mean attention homophily of 0.812 indicates that the encoder preferentially propagates information across biologically similar neighbours, thereby structuring the representation before variational compression. This neighbourhood-aware encoding yields lower intra-cluster variance, higher inter-cluster separation, and a more stable posterior geometry for both NB reconstruction and IB compression.

GAHIB also leads on DRE-local metrics ( $Q_{\text{local}}$ , distance correlation), reflecting that the neighbourhood structure of the cell graph is faithfully preserved in the latent space. Cells that are adjacent in the 15-NN graph retain similar latent positions, so graph topology is encoded as continuous local proximity rather than as a purely *post hoc* clustering signal.

This result underscores a broader principle: explicitly encoding cell-neighbourhood structure during representation learning can provide a stronger inductive bias than posterior regularisation or geometric losses applied *post hoc*. Accordingly, future single-cell VAEs may benefit from prioritising graph-aware encoders before introducing increasingly elaborate latent-space regularisers.

### 8.3 Information bottleneck–hyperbolic coupling

A central architectural property of GAHIB is the *functional coupling* between the information bottleneck and the hyperbolic geometry loss. The IB layer compresses the d -dimensional latent code z into a 2D manifold coordinate $z^{\prime }$ ; this compressed representation then serves as the *anchor* for the Lorentz distance in Equation 2. Without this anchor, the hyperbolic loss operates on an untrained target and becomes degenerate. The ablation confirms this directly: VAE + Hyp (without IB) performs nearly identically to the Base VAE on clustering (NMI 0.653 vs. 0.651), whereas VAE + IB + Hyp recovers the full DRE and LSE benefit (DRE 0.735, LSE 0.534). IB enables the hyperbolic term, while the hyperbolic term then imposes hierarchical organisation on the compressed manifold. This reciprocal interaction provides a principled route for incorporating geometric inductive bias into deep generative models.

### 8.4 Hyperbolic geometry captures hierarchy-related signals

The interpretation study (Section 6) provides quantitative evidence that GAHIB’s Lorentz norm is associated with developmental hierarchy. Stemness–norm Spearman correlations are positive and statistically significant (p<0.001) in the majority of datasets, with the strongest associations observed in developmental systems (dentate gyrus: $\rho_{s}=0.673$ ; breast cancer: 0.615; colorectal adenocarcinoma: 0.505; Merkel cell carcinoma: 0.501). The Poincaré disk projections (Figure 13) provide corresponding visual support: stem and progenitor populations cluster near the disk origin, while differentiated lineages radiate toward the boundary.

The biological rationale for this behaviour follows from the geometry of the Lorentz hyperboloid (Ganea et al., 2018). The origin of hyperbolic space is the unique point of minimum norm; in tree embeddings (Nickel and Kiela, 2017)—and in their single-cell analogue Poincaré maps (Klimovskaia et al., 2020)—the root of the hierarchy maps to the origin while leaves map to high-norm positions. Earlier hyperbolic variational autoencoders (Mathieu et al., 2019; Nagano et al., 2019) established the reparameterisation machinery on the Poincaré disk and the Lorentz model but did not couple this geometry to graph-based encoding or to an information bottleneck. GAHIB’s geodesic loss encourages the high-dimensional latent code z and its 2D projection $z^{\prime }$ to occupy consistent positions on the hyperboloid, effectively regularising the encoder to produce representations whose *radial distance from the origin encodes developmental maturity*. Stem and progenitor cells, which transcriptionally resemble the common ancestor of many lineages, are naturally placed near the origin; their descendants, which have progressively narrowed gene-expression programmes, are pushed toward the boundary. These patterns emerge without pseudotime labels, lineage trees, or any prior knowledge of developmental order.

The Lorentz geometry loss therefore supports a biologically coherent radial ordering of cell states and yields a continuous latent coordinate that correlates with canonical stemness markers on annotated datasets as a by-product of the variational objective.

### 8.5 Disentanglement regularisation hurts single-cell clustering in this benchmark

In the disentanglement benchmark (Section 5.6), every regulariser tested ( β -VAE, DIP-VAE, TC-VAE, InfoVAE) *degrades* clustering quality relative to the unregularised Base VAE, with TC-VAE producing the most severe collapse (NMI 0.404, − 0.243 vs. Base VAE).

Disentanglement objectives penalise statistical dependence among latent dimensions, encouraging a posterior distribution whose marginals are as independent as possible (Higgins et al., 2017; Chen et al., 2018). In natural images, this is beneficial because independent factors (shape, colour, and pose) genuinely exist and each dimension can specialise. In scRNA-seq data, however, the dominant sources of variation are co-regulated gene programmes—transcription factor networks, cell-cycle machinery, lineage-specific regulons—which are fundamentally *correlated* by construction. Forcing the encoder to represent these programmes in orthogonal dimensions requires sacrificing reconstruction fidelity (higher ELBO cost), which degrades the quality of the latent code for all downstream tasks.

InfoVAE (Zhao et al., 2019), which relaxes the factorisation constraint, is almost neutral (NMI 0.649 vs. Base 0.647), consistent with this interpretation: minimal factorisation pressure preserves correlation structure. GAHIB’s IB layer, by contrast, does not impose independence. It imposes a *geometric* constraint—a compact 2D projection that still reconstructs the input—which selects for a low-dimensional manifold without disrupting the correlation structure of the full 10D latent code. Geometric compression rather than statistical independence is what allows GAHIB sustain both strong clustering (NMI 0.722) and high-fidelity dimensionality reduction (DRE 0.700).

### 8.6 Biological interpretability as a design principle

GAHIB’s architectural choices produce directly interpretable biological signals. The GAT encoder’s attention weights encode cell-type homophily (mean 0.812), indicating that the model assigns greater influence to same-type neighbours without explicit supervision. The Lorentz norm’s correlation with stemness ( $\rho_{s}=0.329$ mean; up to 0.673 in dentate gyrus) further demonstrates that hyperbolic geometry captures the continuous nature of differentiation rather than treating it as a discrete cluster assignment. Together, these observations indicate that inductive biases aligned with known biological structure can yield unsupervised biological alignment without requiring cell-type labels during training.

### 8.7 Latent dimensions reflect context-specific biology

Gene Ontology biological process enrichment on four tissue contexts (Section 6.6) provides one stringent biological test of GAHIB’s latent space: when trained independently on each dataset, the model recovers tissue-relevant programmes without apparent cross-contamination. The brain-derived dentate gyrus model returns gliogenesis, neurogenesis, and CNS development (adj. $p\leq 10^{-14}$ ), the hematopoietic model returns leukocyte migration, hemopoiesis, granulocyte activation, and so on, each with the relevant gene markers appearing as expected (Sox9 and Olig2 for glia; Spi1 and Elane for myeloid cells). A latent dimension from the brain-trained model was not observed to be enriched for blood biology, and vice versa—a compositional property that disentanglement regularisers do not achieve because they optimise statistical independence, not biological meaningfulness. This result supports the view that the combined GAT + IB + Hyperbolic framework yields latent factors that are simultaneously (i) statistically informative for clustering and (ii) biologically coherent to the trained tissue.

### 8.8 Reproducibility, cost, and practical deployability

Multi-seed experiments (Section 7.1) show that GAHIB’s clustering performance varies by less than 3% across five random seeds, so the reported gains are not a consequence of selective seed choice. Hyperparameter sensitivity (Section 7.4) reveals that only $\lambda_{\text{hyp}}$ has a meaningful effect on performance (NMI spread Δ=0.044 ), and even this effect is monotonic and can be adjusted; all four hyperparameter ranges include the default value within the observed low-variation range. Computational cost (Section 7.2) shows 22 s per dataset on a mid-range RTX 4080 Laptop GPU with 0.11 GB peak memory—a 41% overhead over Base VAE for the observed benchmark gains. Wall-clock time is flat with cell count in the tested subsampling range (the training loop samples a fixed number of subgraphs per epoch), and per-epoch time is also flat across mini-batch sizes from 32 to 512. These results suggest that GAHIB does not require extensive batch-size tuning and has a practical path toward larger atlas-scale settings with appropriate graph-sampling extensions.

### 8.9 Limitations and future directions

#### 8.9.1 Computational cost

GAHIB requires construction of a k -NN graph and performing GAT message passing, adding approximately 50% wall-clock overhead relative to an MLP-based VAE. For interactive exploratory analysis on small cohorts, this is negligible; for large production pipelines, graph construction can be pre-computed once and cached.

#### 8.9.2 Scalability

Our evaluation caps at 3,000 cells per dataset to ensure fair comparison across all methods. Scaling to atlas-level data ( > 100 K cells) would require efficient graph-sampling strategies such as GraphSAINT (Zeng et al., 2020) or Cluster-GCN (Chiang et al., 2019) combined with mini-batch graph construction, which we leave to future studies. The subgraph sampling strategy we have already employed (10 subgraphs of 512 nodes per epoch) provides a natural foundation for such extensions.

#### 8.9.3 Classical DR competitiveness on linear data

PCA achieves near-identical NMI/ARI to GAHIB on several datasets with linearly separable structure (e.g., NMI 0.735 vs. 0.721), indicating that GAHIB’s advantage is largest for datasets with nonlinear developmental or tumour heterogeneity. Future research could explore hybrid approaches that adaptively select between linear and graph-based encoders based on intrinsic dimensionality estimates.

#### 8.9.4 Leiden proxy labels and circularity

The 53-dataset benchmark uses Leiden-derived labels as a consistent unsupervised reference. This enables broad paired comparison, but it also introduces circularity: representation methods are judged against a graph clustering target derived from the same preprocessed expression matrix. Consequently, we describe NMI/ARI as proxy-label metrics, not as definitive biological accuracy. The curated-label pilot partially addresses this limitation on three annotated systems, but it is too small to replace the broad Leiden-proxy benchmark.

#### 8.9.5 Curated-label coverage

Only three datasets in the available manifests contain curated biological labels suitable for non-Leiden evaluation, and three more contain proxy trajectory or cluster annotations. The remaining 47 datasets lack curated labels in the revised dataset package. Stronger biological validation will require the systematic curation of source annotations or new benchmark datasets with manually verified cell identities and lineage states.

#### 8.9.6 Count-level dropout robustness

The added dropout experiment is a bounded pilot, not a comprehensive robustness claim. It masks non-zero counts in three annotated systems at 0%, 20%, 40%, and 60% and evaluates three methods under a reduced 20-epoch setting. The results show context-dependent degradation rather than universal robustness. A full dropout study should include more datasets, repeated seeds, simulator-based protocols, and matched training budgets.

#### 8.9.7 Metadata provenance

The metadata inventory records available accession/source IDs, cell/gene counts, broad domain, inferred species/tissue strata, and annotation availability. Platform, original count units, source cluster counts, and detailed QC/normalisation provenance remain unavailable for a subset of datasets and are not imputed. Consequently, stratified claims by platform or preprocessing protocol are deferred to future research.

## 9 Conclusion

We present GAHIB, a graph-attention VAE with a hyperbolic information bottleneck for single-cell latent representation learning. The model combines GAT encoding, information bottleneck compression, and Lorentz hyperbolic geometry in a single framework, with the bottleneck providing the target used by the hyperbolic term.

Across 11 experimental studies on 53 scRNA-seq datasets, we demonstrate the following.

1. Each component contributes to performance, with the GAT encoder providing the largest gain and the IB layer being architecturally necessary for hyperbolic geometry.
2. In the Leiden-proxy benchmark, GAHIB provides a balanced aggregate profile against seven published deep-learning methods, three graph-attention controls, five classical DR, five geometric-VAE, and four disentanglement baselines, while key comparisons remain statistically close or context-dependent (notably scVI on ARI and scDHMap/graph-attention controls on DRE-UMAP).
3. Biological interpretation is supported most strongly on annotated or marker-anchored systems: the Muscle atlas case study shows lineage-aligned marker enrichment, Lung-Dev is mixed, and the T-cell immune-subtype task favours scVI. Across the broad diagnostic cohort, hyperbolic radii correlate with stemness ( $\rho_{s}=0.329$ mean across 53 datasets, up to 0.673 on developmental systems), decoder Jacobians recover 31.5% of marker genes, and GAT attention shows 81.2% mean cell-type homophily (46/53 datasets above 0.75).
4. GAHIB shows low variation across repeated runs and tested tuning settings: performance varies by less than 3% across five random seeds, NMI spread is ≤0.044 across the hyperparameter sweeps, and training time is 22 s/dataset with near-flat measured cell-count scaling under the fixed subgraph-sampling settings.

Taken together, these results indicate that coupling graph-structured encoding with hyperbolic geometry through an information bottleneck is a useful design for single-cell latent representation learning when lineage-oriented geometry and neighbourhood preservation are desired. More broadly, the study suggests that neighbourhood structure and hierarchical geometry can be treated as complementary, rather than competing, inductive biases in future single-cell generative models.

## Data Availability

*Code*. The core GAHIB library (model, metrics, interpretation utilities), the 11 experiment runners, and the test suite are publicly available under an open-source licence in the project repository (https://github.com/PeterPonyu/GAHIB). The companion reader site summarising the benchmark structure is shown in Supplementary Figure S4 and is available at https://peterponyu.github.io/gahib-site/. The figure-generation and manuscript-preparation materials will be made available with the publication record where permitted. The curated-label, marker-enrichment, dropout-pilot, cost-ANOVA, and metadata-summary tables supporting these analyses are available in the accompanying repository/release materials or from the corresponding authors on reasonable request. *Datasets*. The 53 scRNA-seq datasets are all publicly available from the NCBI Gene Expression Omnibus (GEO) under the following 42 unique accession numbers: GSE98638, GSE115571, GSE117988, GSE120446, GSE120505, GSE123813, GSE123902, GSE124310, GSE130148, GSE132509, GSE138709, GSE142653, GSE143423, GSE144024, GSE145929, GSE148215, GSE148218, GSE149655, GSE155109, GSE163558, GSE165784, GSE165844, GSE167597, GSE168181, GSE183904, GSE189070, GSE189357, GSE192857, GSE213740, GSE222002, GSE222369, GSE225600, GSE225857, GSE226131, GSE226824, GSE228499, GSE235787, GSE247719, GSE253355, GSE262288, GSE275119, and GSE283205. Together with the accessions listed above, the evaluation includes five additional public benchmark datasets obtained from the scDHMap benchmark repository: dentate gyrus, endoderm (endo), hematopoiesis (hemato), Setty HSPC (setty), and lung, bringing the total to 47 distinct data sources covering the 53 preprocessed evaluation datasets (several GEO accessions contain multiple independent subsets, e.g., GSE123813 provides both basal and squamous cell carcinoma cohorts). Preprocessing scripts that reproduce the exact evaluation splits are included in the code repository.
