# Supplementary material for: GAHIB: graph attention VAE with a hyperbolic information bottleneck for biologically structured single-cell representations
Source: Front Genet. 2026 Jun 26;17:1863100. doi: 10.3389/fgene.2026.1863100 (PMC13350447; doi:10.3389/fgene.2026.1863100)
Supplement: Supplementary file 1 [file DataSheet2.pdf]

GAHIB — Supplementary Figures

This document contains four supplementary figures cited in the main text as Supplementary Figures 1–4. They provide supporting detail on latent-dimension robustness and seed reproducibility, computational cost and scaling behaviour, hyperparameter sensitivity, and the public benchmark companion site.

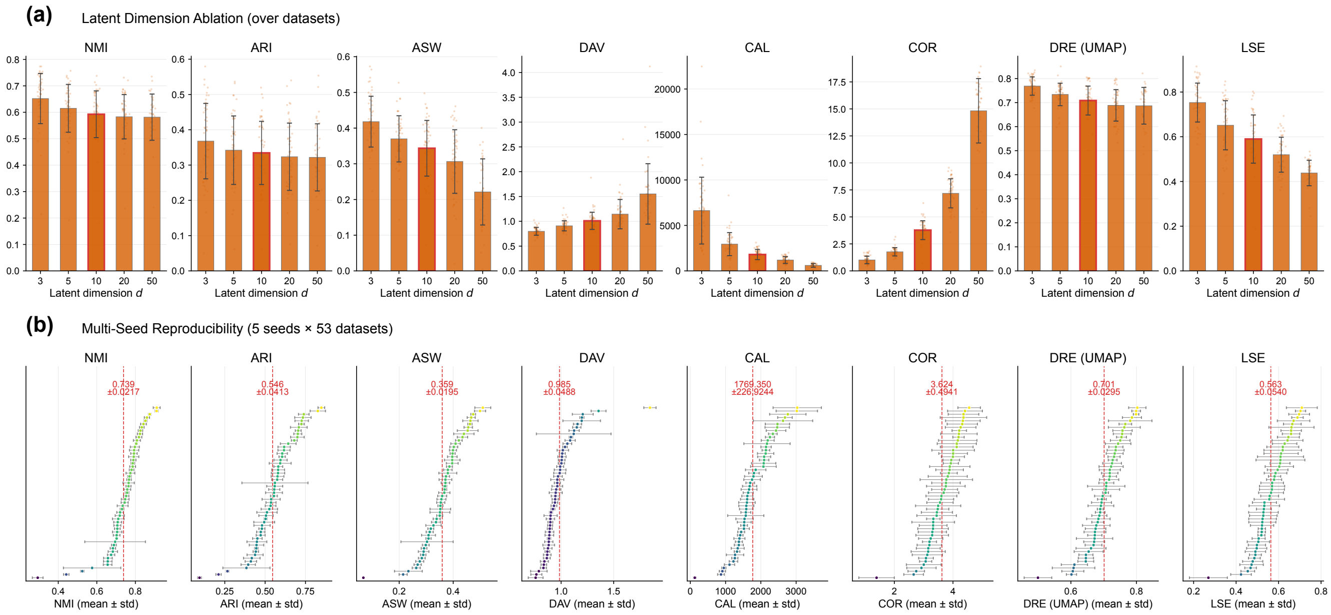

Supplementary Figure 1: Latent dimension ablation and multi-seed reproducibility across 53 datasets, evaluated on eight clustering/embedding metrics (NMI, ARI, ASW, DAV, CAL, COR, DRE (UMAP), LSE). **(a)** Bars show mean per-metric score across 53 datasets as a function of the latent dimension $d \in \{3, 5, 10, 20, 50\}$; error bars denote one standard deviation. The default d=10 (red outline) lies in the low-variation plateau; ASW declines noticeably at d=50. **(b)** Per-dataset multi-seed mean (± std over 5 random seeds); each dot is one dataset, coloured by its mean score, and the red dashed line marks the overall mean.

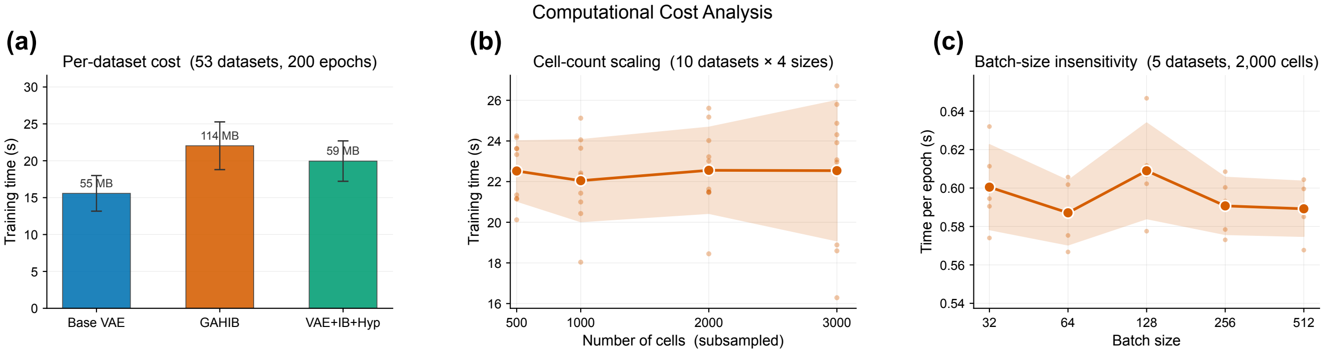

Supplementary Figure 2: Computational cost analysis. **(a)** Training time (bars, mean±std) and peak GPU memory (annotations) for three architectures, aggregated across 53 datasets at 200 epochs; descriptive paired ANOVA values are reported here rather than in a plot inset (time $F_{2,102} = 155.09$, $p = 1.17 \times 10^{-31}$; memory $F_{2,102} = 62057.39$, $p = 4.32 \times 10^{-158}$; actual epochs $F_{2,102} = 1.34$, $p = 0.265$). **(b)** Cell-count scaling: GAHIB training time versus uniform-random subsample size $n \in \{500, 1000, 2000, 3000\}$ cells, measured on 10 representative datasets. **(c)** Batch-size insensitivity at fixed n=2,000 cells across 5 datasets: time per epoch varied by less than 4% across mini-batch sizes $\{32, 64, 128, 256, 512\}$. GAHIB's epoch is paced by a fixed subgraph-sampling schedule, so hardware differences matter less than the sampling cadence. Panels (a) and (b) were measured on an NVIDIA RTX 4080 Laptop GPU; panel (c) used CPU-only execution because sampling overhead, rather than GPU compute, dominates the batch-size comparison.

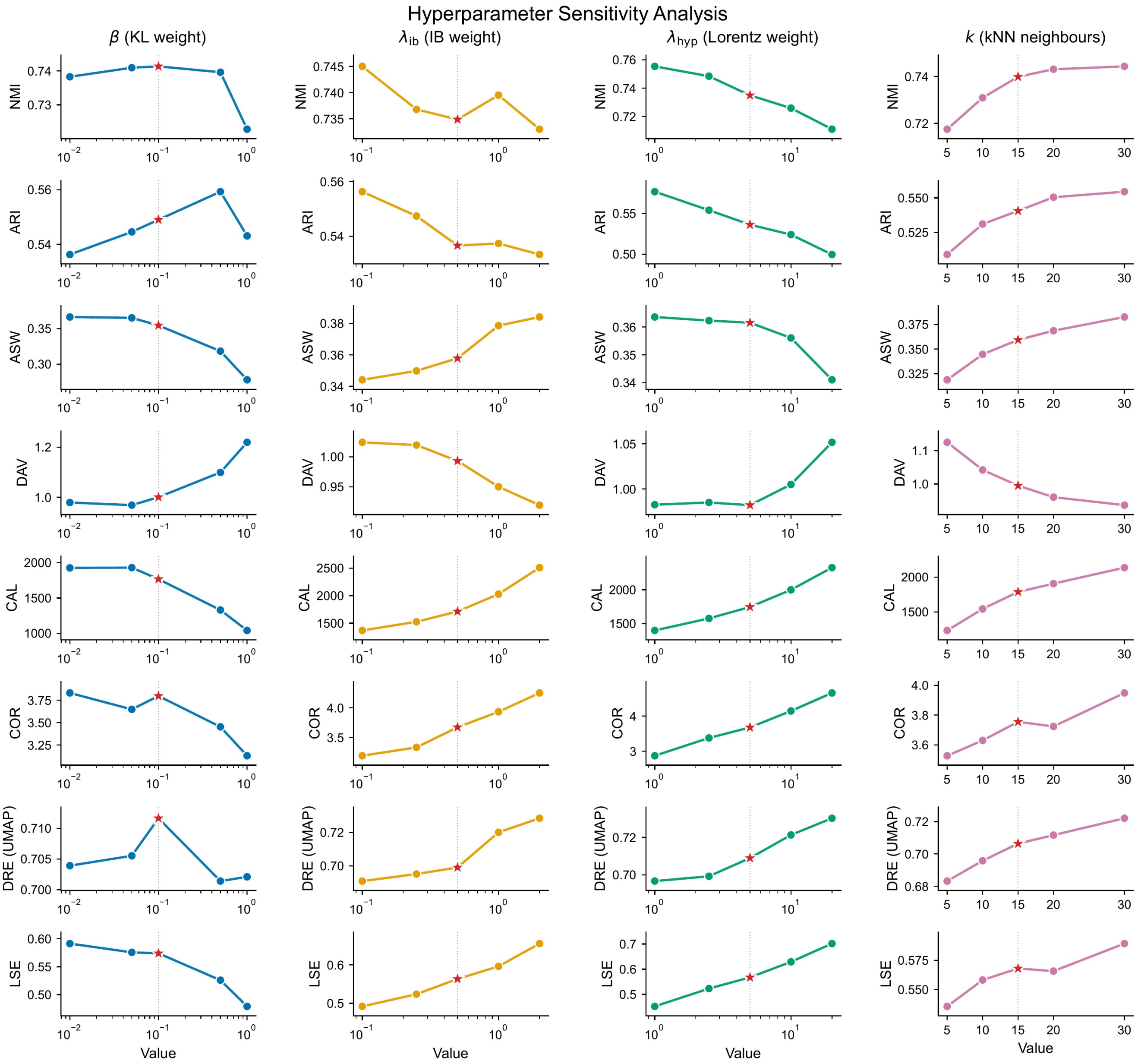

Supplementary Figure 3: Hyperparameter sensitivity across the five key evaluation metrics (NMI, ARI, ASW, DRE UMAP overall quality, LSE overall quality). Each column varies a single hyperparameter while holding others fixed. Red stars indicate the default value used in all other experiments. Scores vary within the plotted ranges; λ_{hyp} exerts the strongest effect on the five metrics.

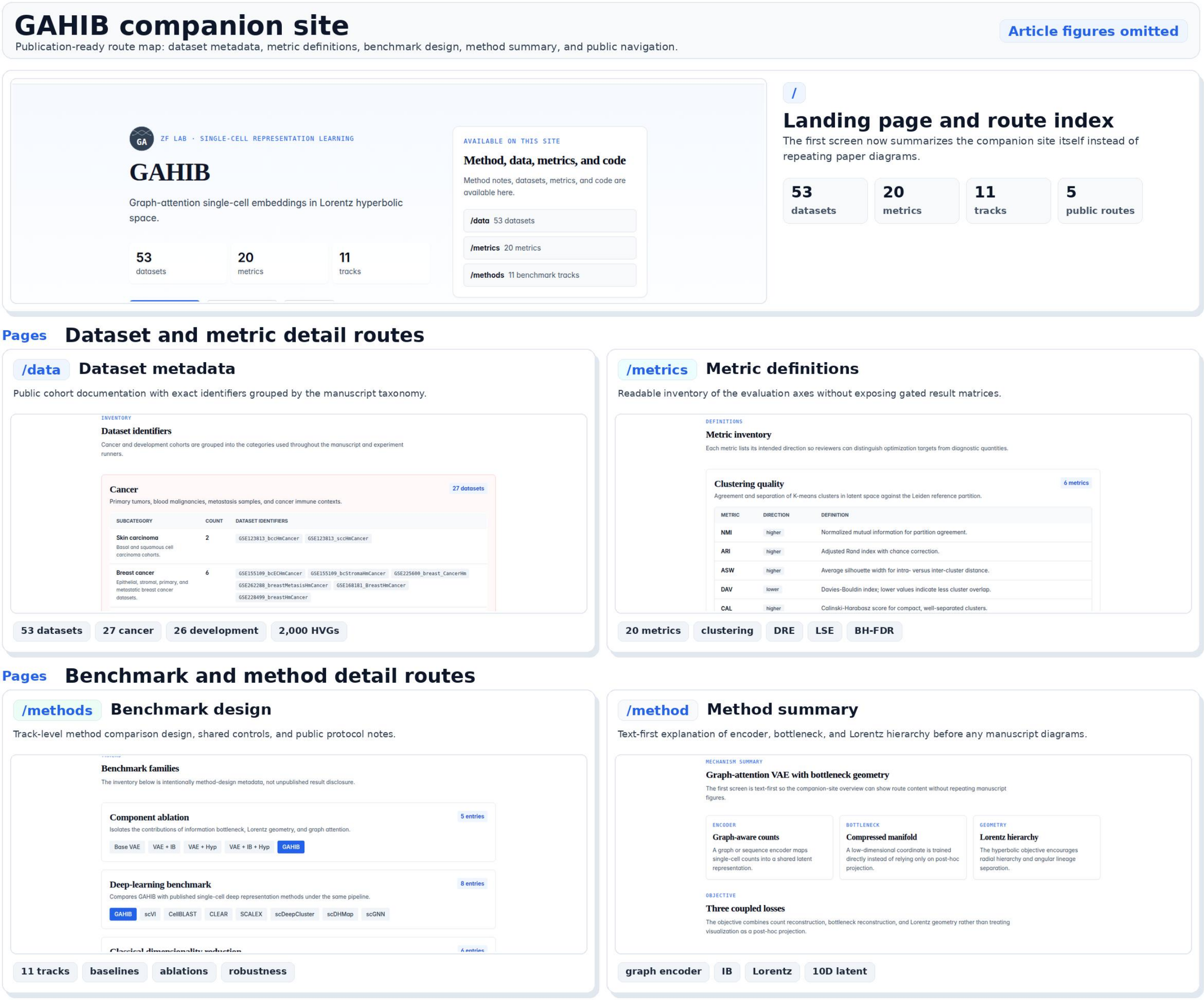

Supplementary Figure 4: Companion reader-site route map for the GAHIB benchmark. Desktop-browser screenshots show the public landing page, dataset inventory, metric definitions, benchmark/method family pages, and GAHIB architecture page. Figure-level article content and quantitative result claims are deliberately omitted from the route cards. This figure serves only as a navigation aid and open-science resource; it does not present or support any additional performance or biological claims.
