# Supplementary material for: GAHIB: graph attention VAE with a hyperbolic information bottleneck for biologically structured single-cell representations
Source: Front Genet. 2026 Jun 26;17:1863100. doi: 10.3389/fgene.2026.1863100 (PMC13350447; doi:10.3389/fgene.2026.1863100)
Supplement: Supplementary file 2 [file DataSheet1.pdf]

GAHIB : Research Highlights & Key Questions

Graph Attention VAE with a Hyperbolic Information Bottleneck
for Biologically Structured Single-Cell Representations

Companion document to the manuscript submitted to *Frontiers in Genetics*.

Research Highlights

The five bullets below summarise the manuscript's central findings and the evidence supporting each.

1 A graph-based hyperbolic single-cell VAE.

GAHIB combines a graph-attention (GAT) encoder, a 2D information bottleneck, and a Lorentz-hyperbolic geometry loss in a single variational objective for scRNA-seq representation learning.

2 Broad benchmark with statistical testing.

Eleven experimental studies on 53 scRNA-seq datasets, evaluated on 20 metrics against 7 deep-learning, 5 classical dimensionality-reduction, 5 geometric-VAE, and 4 disentanglement baselines. GAHIB performs competitively across these baseline families, with BH-corrected improvements in many pairwise comparisons under paired Wilcoxon signed-rank tests.

3 Hyperbolic radii recover developmental hierarchy.

On datasets with lineage annotations, Lorentz norms correlate with developmental order ($\rho_s =$ 0.329 mean, up to 0.673 in enriched contexts; moderate effect), and the resulting pseudotime tracks diffusion pseudotime without using lineage labels during training.

4 Latent dimensions encode tissue-specific biology.

Tissue-specific GO Biological Process enrichment across four tissue contexts confirms that each latent dimension encodes biologically coherent, context-dependent programmes (adj. p down to 10^{-23}) with no off-tissue leakage.

5 Stable across seeds and tuning settings.

Multi-seed runs show $<3\%$ standard deviation across all metrics; robustness sweeps show NMI spread ≤ 0.044 across wide hyperparameter ranges; at fixed 200 epochs, training time is flat with cell count under fixed subgraph sampling.

Key Questions Addressed by This Work

The study addresses five practical questions. Each card below states the question and sketches the evidence the manuscript provides in response.

Zeyu Fu · Army Medical University, Chongqing · fuzeyu99@126.com

Q1 *Can graph attention and hyperbolic geometry be used together in a single VAE, and what does each component contribute?*

Existing single-cell VAEs either use MLP encoders (ignoring cell-graph topology) or explore hyperbolic latent spaces (without neighbourhood-aware encoding). GAHIB combines a GAT encoder, a 2D information bottleneck, and a Lorentz-hyperbolic geometry loss. A component ablation across 53 datasets shows that each part contributes to the overall result ($p < 0.05$, paired Wilcoxon signed-rank, BH-corrected).

Q2 *Does hyperbolic latent geometry genuinely capture developmental hierarchy without explicit supervision?*

Lorentz norms learned by GAHIB align with known differentiation stages ($\rho_s = 0.329$ mean, reaching 0.673 in enriched contexts), producing emergent stemness scores. Pseudotime reconstructed from these norms tracks scanpy's diffusion pseudotime and is validated directly from the underlying gene expression via top $\pm$-correlated HVGs.

Q3 *How does GAHIB compare with leading deep-learning, classical, and geometric-VAE baselines?*

Eleven experimental studies — seven comparative benchmarks against 7 deep-learning, 5 classical DR, 5 geometric-VAE, and 4 disentanglement baselines, plus four robustness/efficiency studies — were conducted on 53 datasets across 6 tissue contexts and 20 evaluation metrics. GAHIB shows consistently strong clustering and dimensionality-reduction fidelity, with statistically supported improvements over many baselines.

Q4 *Are the learned latent dimensions biologically interpretable, and do they capture tissue-specific gene programmes?*

Decoder-Jacobian analysis recovers 31.5% of known marker genes on representative datasets. Critically, GO Biological Process enrichment across four tissue contexts (dentate gyrus, hematopoiesis, Setty HSPC, hESC) shows individual latent dimensions encode distinct, tissue-specific programmes — without any supervised gene-set input during training, and without off-tissue leakage.

Q5 *Is the method stable across seeds and tuning settings, and what is its computational cost?*

Multi-seed experiments (5 runs $\times$ 53 datasets) show <3% standard deviation across all metrics. Hyperparameter-sensitivity analysis confirms stable performance across wide ranges of bottleneck, Lorentz, and KL coefficients. At 200 epochs the per-dataset training time is flat with cell count under fixed subgraph sampling, and requires only a single GPU.

In one sentence. GAHIB combines graph attention, an information bottleneck, and Lorentz-hyperbolic geometry in a single-cell VAE and, across 53 scRNA-seq datasets, yields competitive clustering, dimensionality-reduction fidelity, and lineage-related structure.

Zeyu Fu · Army Medical University, Chongqing · fuzeyu99@126.com
